# Transcriptome analysis of the endangered dung beetle *Copris tripartitus* (Coleoptera: Scarabaeidae) and characterization of genes associated to immunity, growth, and reproduction

**DOI:** 10.1186/s12864-023-09122-w

**Published:** 2023-03-02

**Authors:** Hee Ju Hwang, Bharat Bhusan Patnaik, Snigdha Baliarsingh, Hongray Howrelia Patnaik, Min Kyu Sang, Jie eun Park, Hang Chul Cho, Dae Kwon Song, Jun Yang Jeong, Chan Eui Hong, Yong Tae Kim, Hyeon Jun Sin, Liu Ziwei, So Young Park, Se Won Kang, Heon Cheon Jeong, Hong Seog Park, Yeon Soo Han, Yong Seok Lee

**Affiliations:** 1 Korea Native Animal Resources Utilization Convergence Research Institute (KNAR), Soonchunhyang University, Asan, Chungnam South Korea; 2 PG Department of Biosciences and Biotechnology, Fakir Mohan University, Balasore-, Odisha 756089 India; 3 Department of Biology, College of Natural Sciences, Soonchunhyang University, Asan, Chungnam South Korea; 4 Research Support Center (Core-Facility) for Bio-Bigdata Analysis and Utilization of Biological Resources, Soonchunhyang University, Asan, Chungnam South Korea; 5 iLAB, INSILICOGEN, INC. #2901~2904, Tower-Dong A, HEUNGDEOK IT VALLEY, 13, Heungdeok 1-Ro, Giheung-Gu, Yongin-Si, 16954 Gyeonggi-do Korea; 6 Biodiversity Research Team, Animal & Plant Research Department, Nakdonggang National Institute of Biological Resources, Sangju, Gyeongbuk South Korea; 7 Biological Resource Center (BRC), Korea Research Institute of Bioscience and Biotechnology (KRIBB), Jeongeup, Jeonbuk South Korea; 8 Research Institute, GnC BIO Co., LTD., 621-6 Banseok-Dong, Yuseong-Gu, Daejeon, 34069 Korea; 9 College of Agriculture and Life Science, Chonnam National University, 77 Yongbong-Ro, Buk-Gu, Gwangju, 61186 South Korea

**Keywords:** *C. tripartitus*, Transcriptome, Illumina sequencing, Immunity-related genes, Simple sequence repeats, Informed conservation planning

## Abstract

**Background:**

Dung beetles recycle organic matter through the decomposition of feces and support ecological balance. However, these insects are threatened by the indiscriminate use of agrochemicals and habitat destruction. *Copris tripartitus* Waterhouse (Coleoptera: Scarabaeidae), a dung beetle, is listed as a class-II Korean endangered species. Although the genetic diversity of *C. tripartitus* populations has been investigated through analysis of mitochondrial genes, genomic resources for this species remain limited. In this study, we analyzed the transcriptome of *C. tripartitus* to elucidate functions related to growth, immunity and reproduction for the purpose of informed conservation planning.

**Results:**

The transcriptome of *C. tripartitus* was generated using next-generation Illumina sequencing and assembled de novo using a Trinity-based platform. In total, 98.59% of the raw sequence reads were processed as clean reads. These reads were assembled into 151,177 contigs, 101,352 transcripts, and 25,106 unigenes. A total of 23,450 unigenes (93.40%) were annotated to at least one database. The largest proportion of unigenes (92.76%) were annotated to the locally curated PANM-DB. A maximum of 5,512 unigenes had homologous sequences in *Tribolium castaneum.* Gene Ontology (GO) analysis revealed a maximum of 5,174 unigenes in the Molecular function category. Further, in Kyoto Encyclopedia of Genes and Genomes (KEGG) enrichment analysis, a total of 462 enzymes were associated with established biological pathways. Based on sequence homology to known proteins in PANM-DB, representative immunity, growth, and reproduction-related genes were screened. Potential immunity-related genes were categorized into pattern recognition receptors (PRRs), the Toll-like receptor signaling pathway, the MyD88- dependent pathway, endogenous ligands, immune effectors, antimicrobial peptides, apoptosis, and adaptation-related transcripts. Among PRRs, we conducted detailed in silico characterization of TLR-2, CTL, and PGRP_SC2-like. Repetitive elements such as long terminal repeats, short interspersed nuclear elements, long interspersed nuclear elements and DNA elements were enriched in the unigene sequences. A total of 1,493 SSRs were identified among all unigenes of *C. tripartitus*.

**Conclusions:**

This study provides a comprehensive resource for analysis of the genomic topography of the beetle *C. tripartitus*. The data presented here clarify the fitness phenotypes of this species in the wild and provide insight to support informed conservation planning.

**Supplementary Information:**

The online version contains supplementary material available at 10.1186/s12864-023-09122-w.

## Introduction

Insects are a highly successful taxonomic group, and this success is credited to their physiological plasticity, specifically their fitness genes. In the absence of adaptive immunity, the innate immune system of insects is specialized to provide protection against biotic and abiotic stressors. Insects have habituated to diverse ecosystem types and successfully exploited the complex but dynamic functions of innate immunity to achieve sustained survival. Wild endangered insect species are protected by law and should be prioritized for conservation through elucidation of their fitness phenotypes or adaptation-related transcripts, which could be employed a priori for informed conservation planning [1, 2].

 Dung beetles feed on excrement and play a vital role in the breakdown and recycling of dung into the soil, enabling the nutrients in dung to cycle through the ecosystem. Their utility within tropical forests and agricultural ecosystems is unparalleled [3]. Moreover, these beetles have been introduced into the environment to alleviate ecological damage and maintain ecosystem sustainability [4]. Dung beetle populations have gradually been declining, with several species disappearing and possibly becoming extinct [5–7]. This has alerted conservation biologists to put sustained efforts for genetic rescue of such species in the wild. *Copris tripartitus* (Coleoptera: Scarabaeidae) is a paracoprid dung beetle that feeds on the decaying organic matter and other organic materials in the dung balls. The species has been designated as a class-II endangered species in South Korea [8], but the recent detection of population increases has put an uncertainty to its endangered species status [9]. Widespread efforts are underway to ascertain the population genetic diversity of this insect species to support the development of conservation policies for sustainable protection in wild habitats. With the availability of a mitochondrial genome, genetic analysis of *C. tripartitus* populations initially employed mitochondrial markers such as the cytochrome oxidase I (COI) and cytochrome b (Cytb) genes, and microsatellite markers for this species have been developed recently [10–13]. However, due to the lack of information on the nuclear genome and transcriptome of this species, screening of the fitness phenotypes that influence adaptation to wild habitat perturbations have been difficult. Improving genomics and transcriptomic resources would support the implementation of genetic rescue strategies aiming to re-establish the species in the wild [14]. The development of genomics and transcriptomic resources will provide an atlas of molecular resources that could empower conservation action, while downstream applications based on the functional genomics of immunity, growth, and reproduction-related genes, and the development of microsatellite markers, could inform explicit conservation efforts [14, 15].

In this work, we applied an Illumina-based next-generation sequencing approach (NGS) to develop transcriptome-level molecular resources (i.e., genes associated with growth, immunity, and reproduction) for *C. tripartitus*. In general, transcriptome sequencing is most suitable for the identification of fitness phenotypes associated with immunity, growth, and reproduction in non-model insects, and for the analysis of differential gene expression [16, 17]. Previously, we used an Illumina-based transcriptome approach to assess the physiological attributes of the Asian giant hornet, *Vespa mandarinia* and endangered Lycaenidae butterflies, *Protantigius superans* and *Spindasis takanosis,* thereby supporting conservation actions using genomic resources [1, 18]. The de novo assembled unigenes obtained from the *C. tripartitus* transcriptome were annotated to homologous protein sequences in a locally curated protostome database (PANM-DB) [19]. We screened simple sequence repeats (SSRs) from the coding unigenes, which could be effectively used for studies of polymorphism and population genetics. Molecular resources related to immunity, growth, and reproduction were catalogued and can be accessed as reference data for investigating the plasticity of this species under various habitat-based constraints. Initially, we characterized pattern recognition receptors (PRRs) as immunity genes such as C-type lectin (CTL), peptidoglycan recognition protein (PGRP)-SC2-like, and Toll-like receptor-2 (TLR-2) using a bioinformatics approach. In the context of innate immunity in insects, PRRs such as CTLs, PGRPs and TLRs are indispensable for binding to pathogen-associated molecular patterns (PAMPs) and modulating signal-transduction pathways [20, 21]. The functional data regarding *C. tripartitus* presented in this work could aid decision-making by conservation managers aimed at enhancing its survivability in the wild.

## Methods

### Ethics statement and sample collection

The paracoprid beetle *C. tripartitus* were collected in June 2017 from Seogwipo-si, Jeju-do, Republic of Korea. After transport to the laboratory, the specimens were immediately placed into liquid nitrogen until RNA preparation. This study was undertaken following ethical guidelines for the use of experimental animals in biomedical research [22].

### Total RNA extraction, library construction and Illumina sequencing

The whole body (adult stage) of *C. tripartitus* (*n* = 3) was ground to fine powder in liquid nitrogen using a mortar and pestle. Total RNA was isolated using TRIzol reagent (Invitrogen, Waltham, MA, USA) according to the manufacturer’s instructions, treated with RNase-free DNaseI, and stored at -80 °C until further use. The concentration and purity of the processed RNA sample was determined using a NanoDrop 2000 spectrophotometer (NanoDrop Technologies, Wilmington, DE, USA) and through electrophoresing samples on an agarose gel. The RNA samples were assessed for RNA integrity number (RIN) with an Agilent 2100 Bioanalyzer (Agilent Technologies, Santa Clara, CA, USA). The concentration of RNA was 10.101 ng/µl in a volume of 30 µl, totaling 303.03 ng, and the same RNA was used as the input for library construction and downstream processing. An mRNA-seq library construction kit (Illumina, Inc. San Diego, CA, USA) was used to generate the cDNA library following the manufacturer’s instructions and sequencing was performed using the Illumina HiSeq 4000 (Illumina) NGS platform at GnC Bio-Company (Yuseong-gu, Daejeon, South Korea). Briefly, mRNA was purified from total RNA using oligo(dT) magnetic beads. The refined mRNA was broken into short fragments (200 nt) using an RNA fragmentation kit (Ambion, Austin, TX, USA). The first-strand cDNA was synthesized from mRNA short/cleaved fragments using random-hexamer primers and reverse transcriptase (Invitrogen). The second-strand cDNA was synthesized with RNase H (Invitrogen) and DNA polymerase I (New England BioLabs, Ipswich, MA, USA). After ligating the cDNA to sequencing adapters with paired-end (PE) Adapter Oligo Mix using T4 DNA ligase, purification was accomplished with the QIAquick PCR extraction kit. On the Illumina HiSeq 4000 sequencing platform, DNA fragments (cDNA libraries) of the necessary size (200 ± 25 bp) were sequenced to create 125-bp PE reads. The sequencing reads were then transformed into raw reads through base calling and stored in fastq format. All raw data obtained from sequencing were stored in the National Center for Biotechnology Information (NCBI) Sequence Read Archive (SRA) under accession numbers SRR9951154, BioProject-PRJNA559824, and BioSample-SAMN12560641 (https://www.ncbi.nlm.nih.gov/sra/?term=txid438892[Organism:noexp]).

### Pre-processing of sequencing data and de novo transcriptome assembly

Raw sequencing data were cleaned to remove low-quality reads (> 50% of bases with a Q-value ≤ 20), adapter sequences and ambiguous bases using Cutadpat 1.18 software with the default parameters [23]. FastQC software (version 0.11.5; http://www.bioinformatics.babraham.ac.uk) was used to analyze the quality of the raw reads in fastq format. The clean reads so obtained, were subsequently processed using the Trinity short- read assembly tool [24]with the default setting of 200 bp as the minimum permitted length. The Illumina short-reads were grouped to generate contigs ('Inchworm' assembly phase), which were then clustered and processed to produce a de Bruijin graph ('Chrysalis' phase). All likely sequences were extracted from individual components of the parallelized de Bruijin graphs ('Butterfly' phase). The tool cd-hit-est version 4.6.6 [25] was used to eliminate redundancy from the clustered datasets, and TransDecoder software (version v5.5.0; (https://github.com/TransDecoder/TransDecoder/releases/tag/TransDecoder-v5.5.0) was used to screen for candidate transcript sequences with coding regions.

### Homology search and functional annotation of unigenes

Using BLASTx, the non-redundant unigene sequences were annotated to the locally curated comprehensive protein database PANM-DB (version 3.0) [19]. The Swiss-Prot protein sequence and UniGene nucleotide sequence databases were also searched using BLASTx, with an E-value threshold of 1.0E-5, to obtain homologous sequences. The EuKaryotic Orthologous Groups (KOG) database was searched to enable the classification of unigenes, based on specific functional descriptors, within major categories such as ‘Cellular Processes and Signaling’, ‘Information Storage and Processing’, ‘Metabolism’, and ‘Poorly characterized’ (https://www.ncbi.nlm.nih.gov/COG/). The conserved domains in the unigenes were annotated using the InterProScan (IPS) feature of the BLAST2GO suite (version 5.1) (https://www.blast2go.com). The Gene Ontology (GO) classifications (at level 2) of *C. tripartitus* unigenes (E-value threshold of 1.0E-5) were plotted on a clustered bar chart in Microsoft Excel (Microsoft Corp., Redmond, WA, USA) with the categories such as ‘biological process’, ‘cellular component’, and ‘molecular function’. The Kyoto Encyclopedia of Genes and Genomes (KEGG) database was used for pathway analysis of unigene sequences. (http://www.genome.jp/tools/kaas/).

### Gene discovery related to immunity, reproduction, and growth

Candidate genes associated with immunity, reproduction, and growth were screened using a keyword search of the BLASTx-annotated PANM-DB. The names of representative genes involved in various stages of insect immunity, cell signaling, sex-determination, reproduction and growth processes were included as keywords. Further, the GO terms and KEGG classifications were also referred to identify putative functional transcripts. A comprehensive network of immunity-related transcripts were screened and was categorized into ‘Pathogen Recognition Receptor (Immune signaling pathway)’, ‘TLR Signaling Pathway (Adapter proteins, MyD88-dependent pathway)’, ‘Endogenous Ligands’, ‘Immune Effectors’, ‘Antimicrobial Peptides’, ‘Cytokines and Cytokine Receptors’, ‘Apoptosis’ and ‘Autophagy’.

### Bioinformatics analysis

We conducted an open reading frame (ORF) prediction analysis for selected assembled unigene sequences putatively identified as TLR-2, CTL, and PGRP-SC2-like using the hidden Markov model (HMM)-based FGENESH program (http://www.softberry.com/berry.phtml?topic=fgenesh&group=programs&subgroup=gfind). The predicted ORF sequences were used as the query against the NCBInr database to obtain homologous sequences. After validation, the translated amino acid sequences were used as queries for predictive analyses of protein sequence and structure. The predicted ORF sequences were formatted using the text editor UltraEdit64-bit. SignalP (http://www.cbs.dtu.dk/services/SignalP/) was used to determine the presence of signal peptides. Transmembrane regions were predicted using TMHMM Server v.2.0 (http://www.cbs.dtu.dk/services/TMHMM/). The domain architecture of the protein sequences was retrieved using the SMART domain analysis program at http://smart.embl-heidelberg.de/. Secondary structure prediction was conducted using the program PSIPRED (http://bioinf.cs.ucl.ac.uk/psipred/). Multiple sequence alignments were performed using the program ClustalX2 (version 2.0) [26]. The phylogenetic tree was constructed using the maximum-likelihood method with the 1,000 bootstraps. The phylogenetic tree was visualized using the Molecular Evolutionary Genetics Analysis (MEGA) suite (ver. 11.0) (https://www.megasoftware.net/) [27].

### Identification of repeats and microsatellite marker discovery

The Perl script program MicroSAtellite (MISA) (http://pgrc.ipk-gatersleben.de/misa/) was used to detect SSRs from C. tripartitus unigenes, that were classified into di-, tri-, tetra-, penta-, and hexanucleotide repeats. In the homology-based repeat search process, mononucleotide repeats were excluded from the analysis because Illumina sequencing induces homopolymer formation. RepeatMasker (ver. 4.0.6) was used to screen for representative repeats such as ‘Short Interspersed Nuclear Elements (SINEs)’, ‘Long Interspersed Nuclear Elements (LINEs)’, ‘Long Terminal Repeat (LTR) elements’, and ‘DNA elements’ (http://ftp.genome.washington.edu/RM/RepeatMasker.html). In this process, small RNAs, satellites, simple repeats, and low-complexity repeating elements were screened from the assembled unigene sequences.

## Results

### Illumina sequencing and de novo assembly

**Fig. 1:**
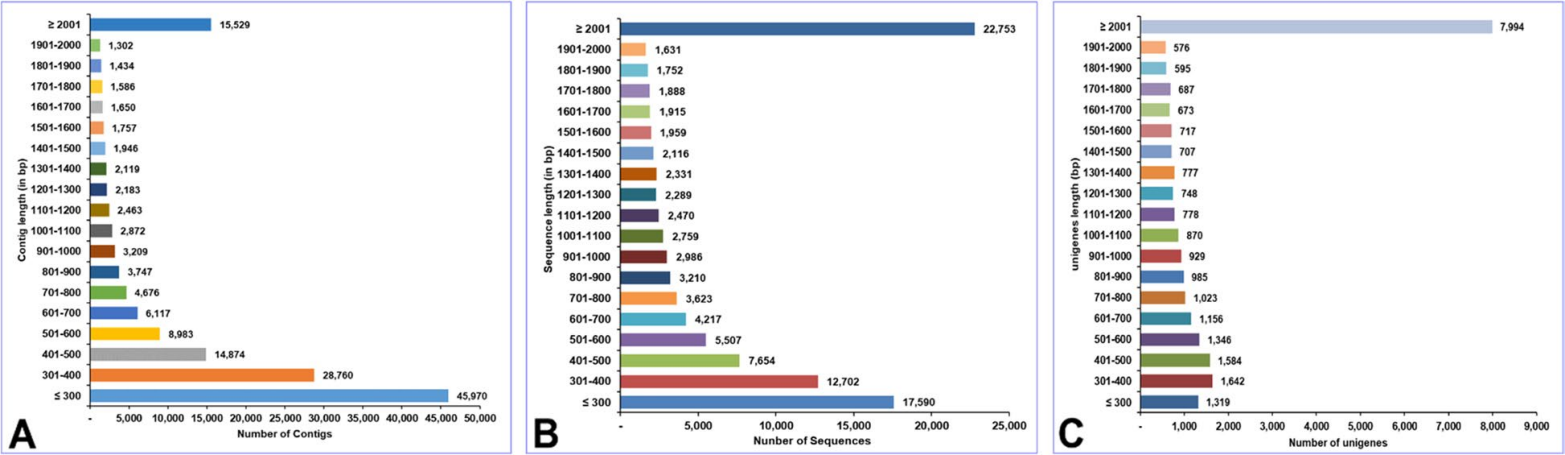
Size distribution of de novo assembled sequences obtained from the *C. tripartitus* transcriptome using the Illumina HiSeq4000 NGS platform. Clean reads were clustered using the Trinity short-read assembly tool to generate contigs. TransDecoder was used to identify the coding transcript sequences, followed by clustering of the datasets into unigenes. **A** Contig length distribution, (**B**) non-redundant sequences length distribution, and (**C**) unigene length distribution

The Illumina short read sequencing platform was utilized to obtain PE reads (25,603,641 × 2 = 51,207,282 raw read sequences; 7,157,952,349 bases). The raw read sequences were pre-processed and 99.84% of sequence bases were retained, with an average length of 139.6 bp (Table S1). In total, 98.58% of the raw read sequences (96.63% of bases) were processed as clean reads. The mean length, N50 length, and GC content of clean read sequences was 137 bp, 151 bp, and 40.42%, respectively. De novo assembly of clean reads generated a total of 151,177 contigs (127,555,512 bases) with an average size of 843.7 bp (largest contig size, 42,685 bp). Overall, 40.81% and 23.07% of the contig sequences had sizes of ≥ 500 and ≥ 1,000 bp, respectively. The TransDecoder program identified 67.04% of all contig sequences as likely to contain coding regions. The mean length, N50 length, and GC content of TransDecoder-derived sequences was 1,392.9 bp, 2,469 bp, and 38.72%, respectively. Approximately, 62.62% and 43.31% of the sequences had lengths of ≥ 500 and ≥ 1,000 bp, respectively. Clustering of sequences with potential coding regions using the TGICL tool identified 25,106 unigenes (45,071,628 bases). The mean length, N50 length, and GC% of the unigenes were 1,795.3 bp, 2,667 bp, and 38.54%, respectively. The unigenes ranged from 224 to 43,765 bp in length. Approximately, 81.95% and 60.26% of unigenes showed lengths of ≥ 500 and ≥ 1,000 bp, respectively. A statistical summary of the de novo assembled transcriptome of *C. tripartitus* is provided in Table 1. Figure 1 shows the distributions of contigs, sequences and unigenes based on their sizes. Only 10.27% and 30.41% of contig sequences had lengths of ≥ 2,001 and ≤ 300 bp, respectively (Fig. 1A). Further, 22.44% and 17.35% of TransDecoder-derived sequences were ≥ 2,001 and ≤ 300 bp, respectively. (Fig. 1B). In total, 31.84% of the total unigenes had lengths of ≥ 2,001 bp, which increased the feasibility of obtaining full-length transcripts (Fig. 1C). In summary, a greater number of unigenes with length of ≥ 2,001 bp were obtained.

**Table 1:** Statistical summary of *C. tripartitus* transcriptome

Total number of clean reads	
- Number of sequences	50,482,764
- Number of bases	6,917,061,418
- Mean length of clean reads (bp)	137.0
- N50 length of clean reads (bp)	151
- GC % of contig	40.42
High-quality reads (%)	98.58 (sequences), 96.63 (bases)
Contig information	
- Total number of contig	151,177
- Number of bases	127,555,512
- Mean length of contig (bp)	843.7
- N50 length of contig (bp)	1,593
- GC % of contig	37.97
- Largest contig (bp)	42,685
- No. of large contigs (≥ 500 bp)	61,695
- No. of large contigs (≥ 1,000 bp)	34,874
TransDecoder information	
- Total number of sequences	101,352
- Number of bases	141,178,205
- Mean length of sequence (bp)	1,392.9
- N50 length of sequence (bp)	2,469
- GC % of sequence	38.72
- Largest sequence (bp)	42,685
- No. of large sequences (≥ 500 bp)	63,464
- No. of large sequences (≥ 1,000 bp)	43,896
Unigene information	
- Total number of unigenes	25,106
- Number of bases	45,071,628
- Mean length of unigene (bp)	1,795.3
- N50 length of unigene (bp)	2,667
- GC % of unigene	38.54
- Length ranges (bp)	224—43,765
- No. of large unigenes (≥ 500 bp)	20,574
- No. of large unigenes (≥ 1,000 bp)	15,129

### Sequence annotation and functional gene enrichment analysis

**Fig. 2:**
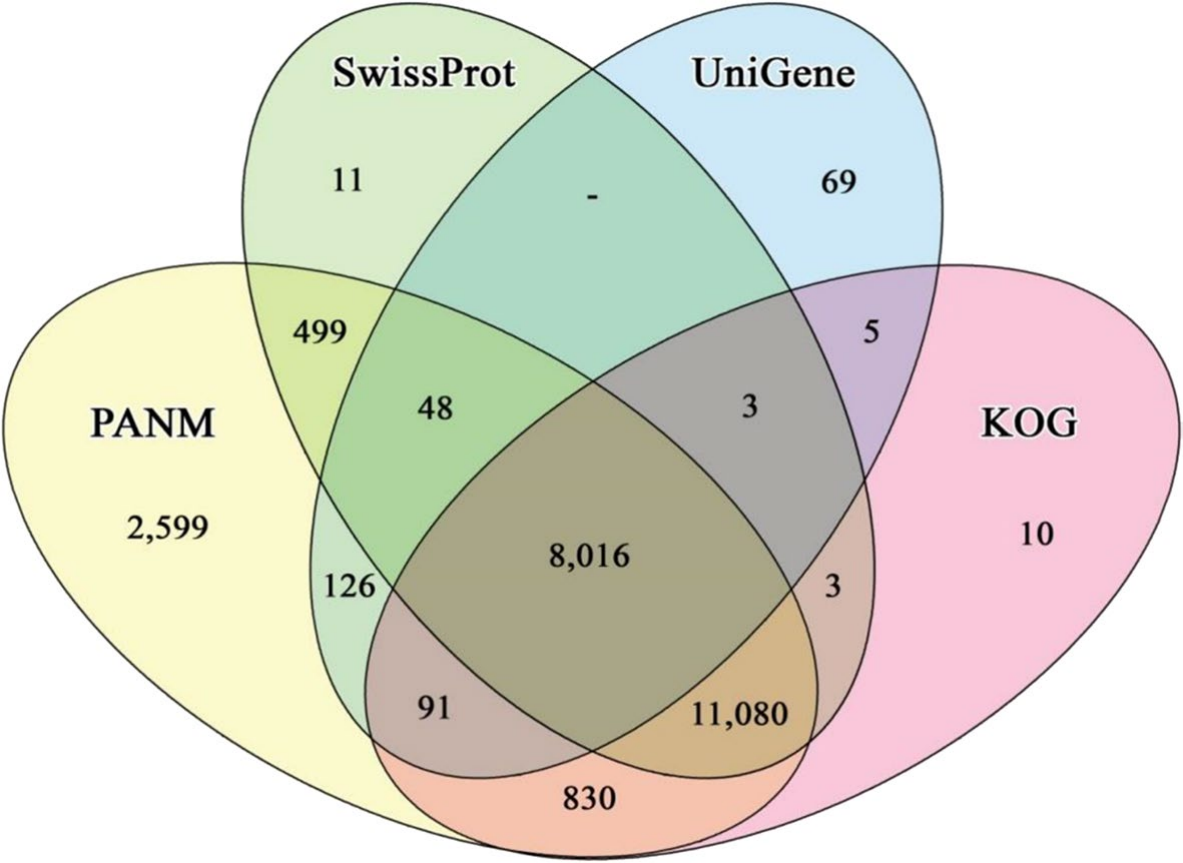
Annotation of *C. tripartitus* unigenes against public protein and nucleotide databases (PANM-DB, Swiss-Prot, UniGene, and KOG). Venn diagram showing homologous matches of unigenes to the selected databases (specific or overlapping)

The sequence annotation statistics (Table 2) indicated that out of the 25,106 de novo assembled unigenes, 93.40% matched homologous sequences across all databases. In total, 92.7% of all unigenes showed homologous matches to sequences in PANM–DB, followed by 79.81%, 78.30%, 56.71%, 53.95%, 33.29%, and 2.70% in the KOG, Swiss-Prot, GO, IPS, UniGene, and KEGG databases, respectively. Out of the PANM–DB annotated sequences, 64.11% showed lengths of ≥ 1,001 bp. Furthermore, 69.11%, 69.68% and 79.29% of unigenes annotated against the KOG, Swiss-Prot and UniGene databases had lengths of ≥ 1000 bp, respectively. The Venn diagram (Fig. 2) illustrates that a total of 2,599 unigenes annotated to homologous proteins present in PANM–DB. Further, a total of 11,080 unigenes showed matches to homologous proteins in PANM, Swiss-Prot and KOG databases. Furthermore, 8,016 unigenes found homologous matches in all the four databases. This suggests that a greater number of unique matches were represented under PANM-DB.

**Table 2:** Distribution of *C. tripartitus* transcripts under the publicly available databases

	all unigenes	< 500 bp	500–1000 bp	1001–2000 bp	2001–3000 bp	> 3000 bp
PANM-DB	23,289	3,502	4,856	6,976	3,786	4,169
UniGene	8,358	568	1,163	2,278	1,800	2,549
Swissprot	19,660	2,362	3,598	6,128	3,529	4,043
KOG	20,038	2,464	3,725	6,254	3,561	4,034
GO	14,238	1,469	2,467	4,467	2,675	3,160
KEGG	677	36	67	197	169	208
IPS	13,545	1,287	2,306	4,107	2,603	3,242
ALL	23,450	3,566	4,926	6,994	3,789	4,175

**Fig. 3:**
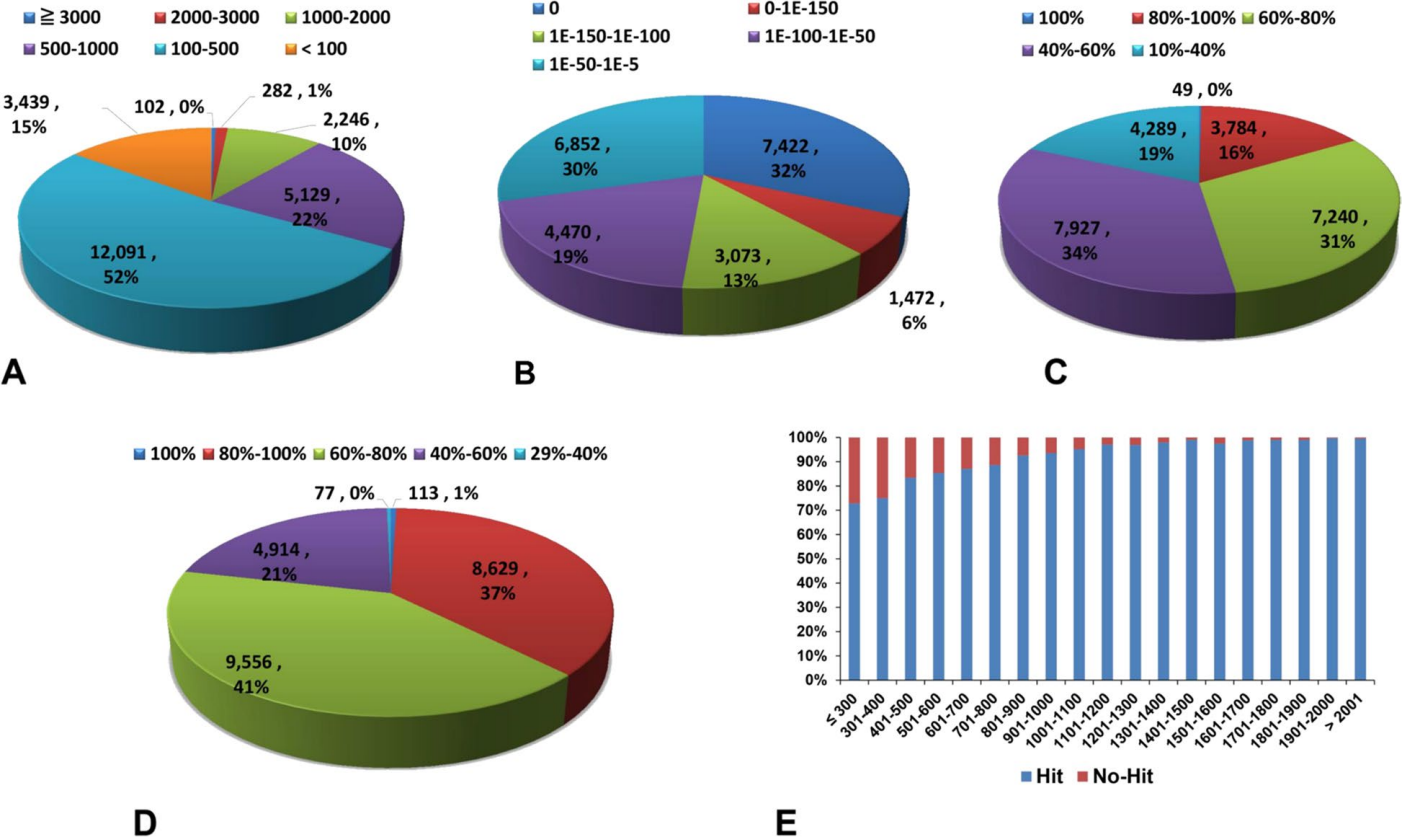
Statistical features of *C. tripartitus* unigenes against PANM-DB. BLASTx annotation of the unigenes to PANM-DB at an E-value threshold of 1.0E-5 was used to obtain the homology statistics. **A** Score distribution, (**B**) E-value distribution, (**C**) identity distribution, (**D**) similarity distribution, and (**E**) sequence hits/non-hits correlated with the length of unigenes

**Fig. 4:**
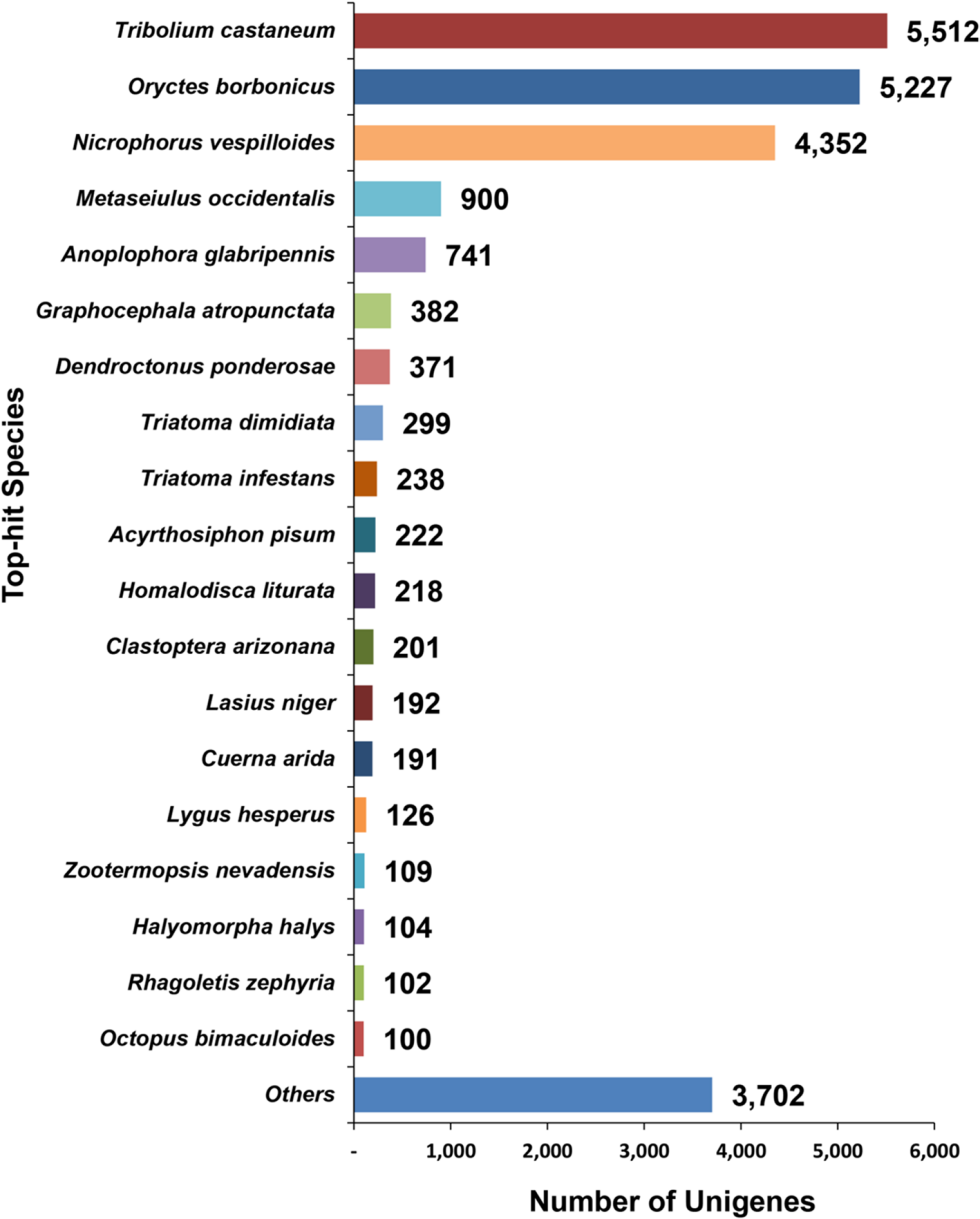
Species distribution of the top matches to *C. tripartitus* unigenes from homologous sequences in PANM-DB (BLASTx; E-value cutoff of 1.0E-5). The strongest matches to unigenes were observed for homologous proteins of *T. castaneum*

The homology matrices for statistical evaluation of the unigenes annotated against PANM–DB using BLASTx analysis have been shown in Fig. 3. The score value distribution revealed that 52% and 22% of unigenes have homology scores of 100–500 and 500–1,000, respectively (Fig. 3A). The E-value distribution revealed a maximum of 32% followed by 30% unigenes showing homology at 0 and 1E-50 to 1E-5, respectively (Fig. 3B). The identity distribution (Fig. 3C) of unigenes shows a total of 34%, followed by 31%, and 19% having identities of 40–60%, 60–80%, and 10–40%, respectively. Only 16% sequences showed 80–100% identity to the homologous sequences in the PANM-DB database. The similarity distribution indicated that 41%, 37%, and 21% of unigenes had similarities of 60–80%, 80–100%, and 40–60%, respectively (Fig. 3D). The number of annotation hits compared to non-hits increased in direct proportion to the length of unigenes (Fig. 3E). A maximum of 7,955 hits (39 non-hits) to homologous sequences in the PANM database had lengths of ≥ 2,001 bp (Fig. 3E). Further, in the top-hit species distribution, a maximum of 23.66% of unigenes were annotated with homologous proteins in the red flour beetle, *Tribolium castaneum*, followed by 22.44% and 18.68% for the scarab beetle *Oryctes borbonicus* and burying beetle *Nicrophorus vespilloides*, respectively. Excepting the mollusc *Octopus bimaculatus* all other representative top-hit species belonged to insects (Fig. 4).

**Fig. 5:**
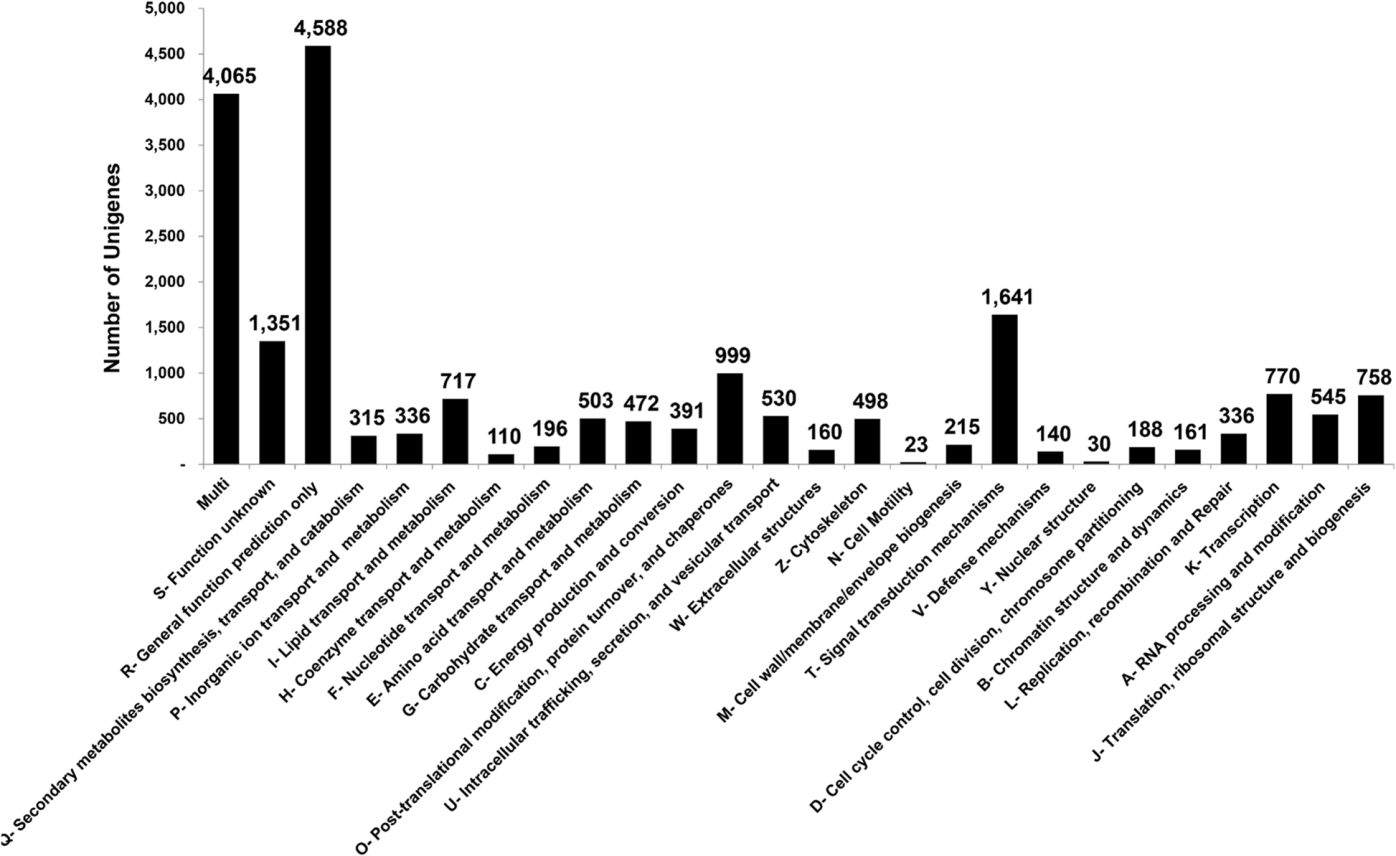
Functional classification of *C. tripartitus* unigenes against the KOG database. Of 25,106 non-redundant unigene sequences, 20,038 were classified into 25 functional KOG categories, excluding the multifunctional category. Most unigenes were classified into the ‘General function prediction only’, ‘Multi’, and ‘Signal transduction mechanisms’ categories

To investigate functional directions, we annotated *C. tripartitus* unigenes against the KOG, GO, KEGG, and IPS databases. The unigenes were annotated under 25 KOG functional categories (excluding the ‘multi’ category). The highest percentage (22.9%) of unigenes was classified as R- ‘general function prediction only’, followed by 20.3% of sequences in ‘multiple’ KOG categories. Approximately, 8.2% and 6.7% of unigenes were classified into the T- ‘signal transduction mechanisms’, and S- ‘function unknown’ categories, respectively (Fig. 5). The least populated KOG functional terms included H- ‘co-enzyme transport and metabolism’, N- ‘cell motility, and Y- ‘nuclear structure’. Further, in the IPS annotation, a maximum of 1,407 unigenes contained the zinc finger C2H2-type domain, followed by 433 and 275 sequences with protein kinase and ankyrin repeat domains, respectively (Table 3). The other prominent domains included the ABC transporter-like domain, immunoglobulin-like domain, EGF-like domain, and small GTP-binding protein domain, that might be prominent in proteins belonging to the innate immune signaling pathways of *C. tripartitus*.

**Table 3:** List of top-30 protein domains found in *C. tripartitus* unigene sequences

Domain	Description	Unigene (no.)
IPR013087	Zinc finger C2H2-type	1407
IPR000719	Protein kinase domain	433
IPR020683	Ankyrin repeat-containing domain	275
IPR000504	RNA recognition motif domain	266
IPR000477	Reverse transcriptase domain	234
IPR020846	Major facilitator superfamily domain	223
IPR001254	Serine proteases, trypsin domain	213
IPR017986	WD40-repeat-containing domain	212
IPR000210	BTB/POZ domain	186
IPR001478	PDZ domain	186
IPR001452	SH3 domain	176
IPR001849	Pleckstrin homology domain	169
IPR001841	Zinc finger, RING-type	163
IPR003593	AAA + ATPase domain	155
IPR002048	EF-hand domain	145
IPR007110	Immunoglobulin-like domain	131
IPR014001	Helicase superfamily 1/2, ATP-binding domain	130
IPR001650	Helicase, C-terminal	124
IPR005225	Small GTP-binding protein domain	120
IPR013026	Tetratricopeptide repeat-containing domain	116
IPR001584	Integrase, catalytic core	109
IPR003439	ABC transporter-like	108
IPR011705	BTB/Kelch-associated	107
IPR000742	EGF-like domain	106
IPR002018	Carboxylesterase, type B	102
IPR003599	Immunoglobulin subtype	101
IPR003961	Fibronectin type III	99
IPR000873	AMP-dependent synthetase/ligase	97
IPR003598	Immunoglobulin subtype 2	97
IPR001251	CRAL/TRIO lipid binding domain	93

**Fig. 6:**
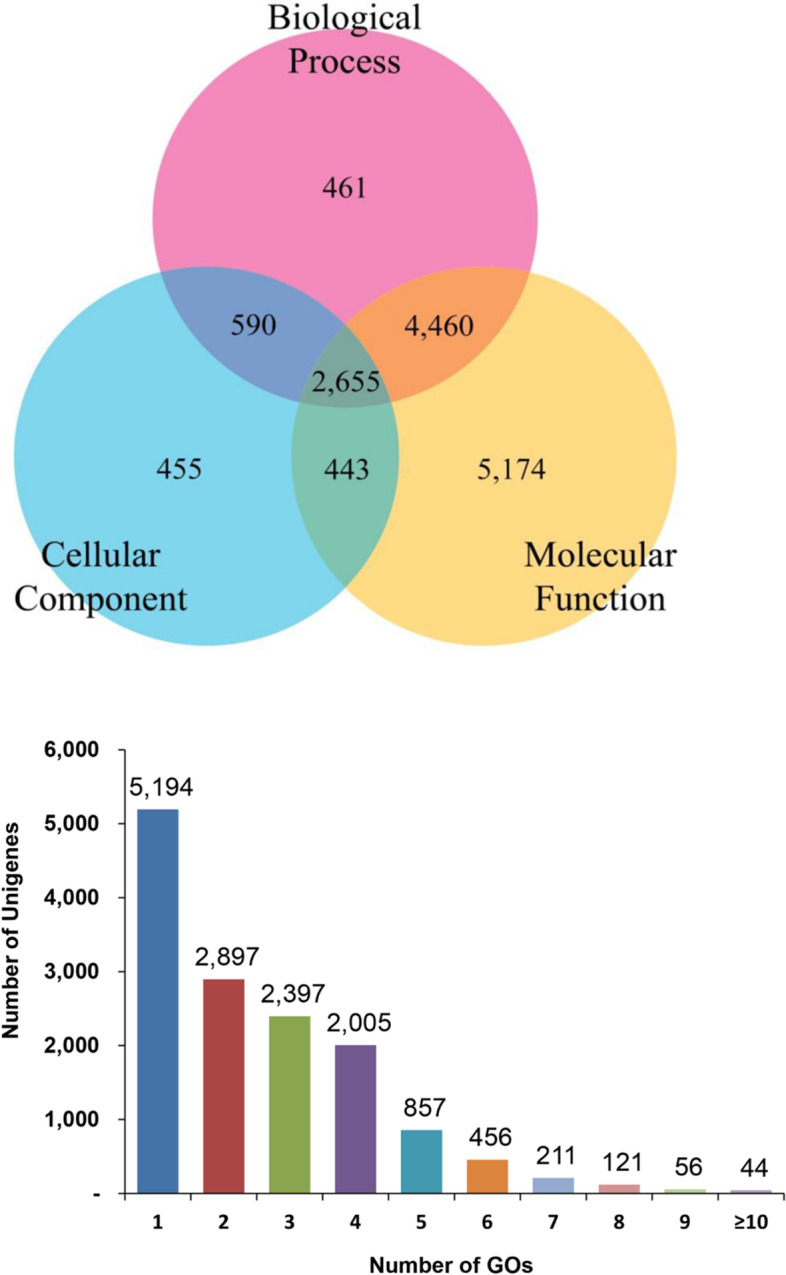
Gene Ontology (GO) assignments of *C. tripartitus* unigenes. **A** Venn diagram showing the distribution of unigenes among the GO functional categories ‘Biological Process’, ‘Cellular Component’, and ‘Molecular Function’. **B** Number of unigenes assigned to various GO terms

**Fig. 7:**
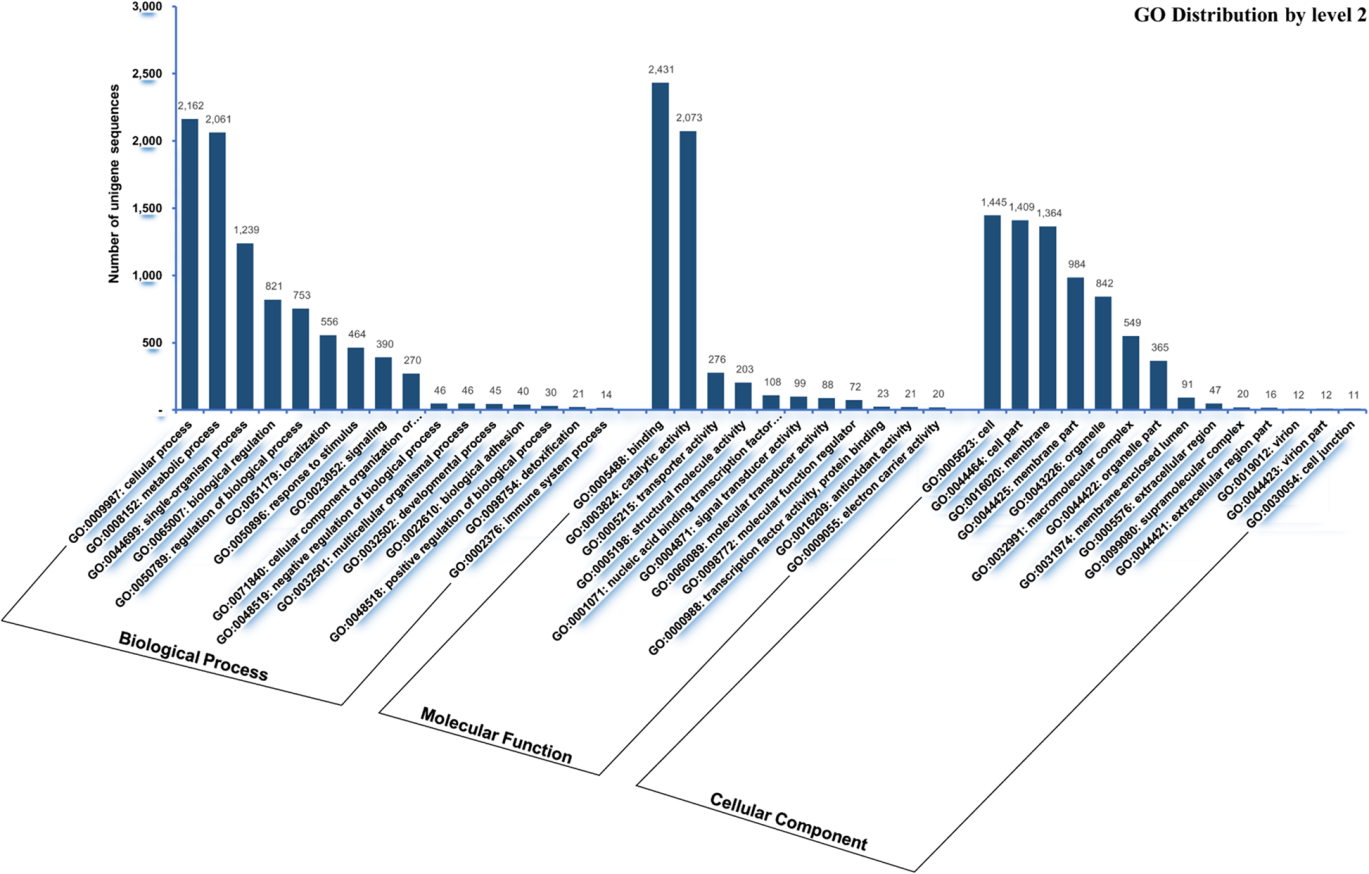
Assignment of *C. tripartitus* unigenes to the GO terms ‘Biological Process’, ‘Cellular Component’, and ‘Molecular Function’. The Y-axis shows the number of unigenes assigned to each GO term at level 2

**Fig. 8:**
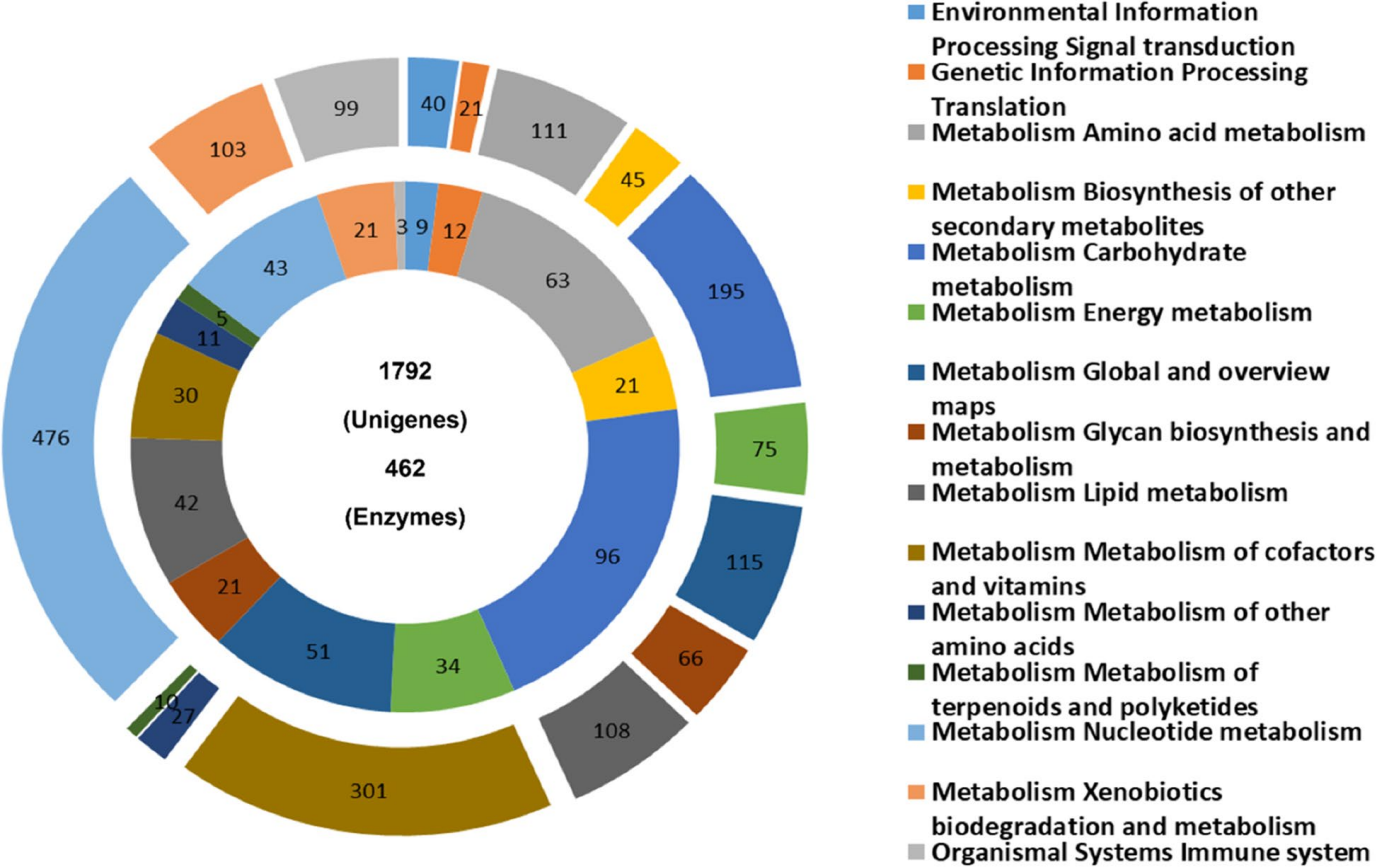
KEGG pathway distribution. The “doughnut graph” shows the number of unigenes encoding enzymes in the pathway (outer circle) and the number of enzymes in the pathway (inner circle). The unigenes and enzymes in the pathway were classified as ‘Metabolism’, ‘Genetic information processing’, Environmental information processing’, and ‘Organismal systems’

A total of 14,238 unigenes were annotated to GO functional categories such as ‘Molecular function’, ‘Biological process’, and ‘Cellular component’ (Fig. 6). A three-way Venn diagram was constructed to illustrate the categorization of unigenes to GO functional categories and their overlap, if any. The largest number of unigenes (*n* = 12,732) were functionally annotated to the ‘Molecular function’ category. A total of 2,655 unigenes were ascribed to all three GO functional categories. In contrast, 5,174, 461, and 455 unigenes were exclusively assigned to the ‘Molecular function’, ‘Biological process’ and ‘Cellular component’ categories, respectively (Fig. 6A). In total, 5,194 unigenes had only one GO term, while 2,897, 2,397, and 2,005 had two, three, and four GO terms, respectively. (Fig. 6B). Figure 7 shows the annotations of unigenes to individual functional GO terms (at level 2) within the three GO functional categories. Within the ‘Biological process’ category, most unigenes were annotated to the cellular process term (GO: 0009987), followed by metabolic process (GO: 0008152), and single-organism process (GO: 0044699). Binding (GO: 0005488) and catalytic activity (GO: 0003824) were the major GO terms within the ‘Molecular function’ category and cell (GO: 0005623), cell part (GO: 0044464), membrane (GO: 0016020), membrane part (GO: 0044425), and organelle (GO: 0043226) were important terms in the ‘Cellular component’ category. The annotation of unigenes based on KEGG pathways revealed enzymes in four functional categories namely ‘Environmental information processing’, ‘Genetic information processing’, ‘Metabolism’, and ‘Organismal system’. In total, 462 putative enzymes were annotated, represented by 1,792 unigenes. Most unigenes predicted to be putative enzymes were assigned to ‘Metabolism’, followed by the ‘Organismal system (immune system)’ category (Fig. 8).

### Characterization of repeating elements and SSRs in *C. tripartitus* transcriptome

**Fig. 9:**
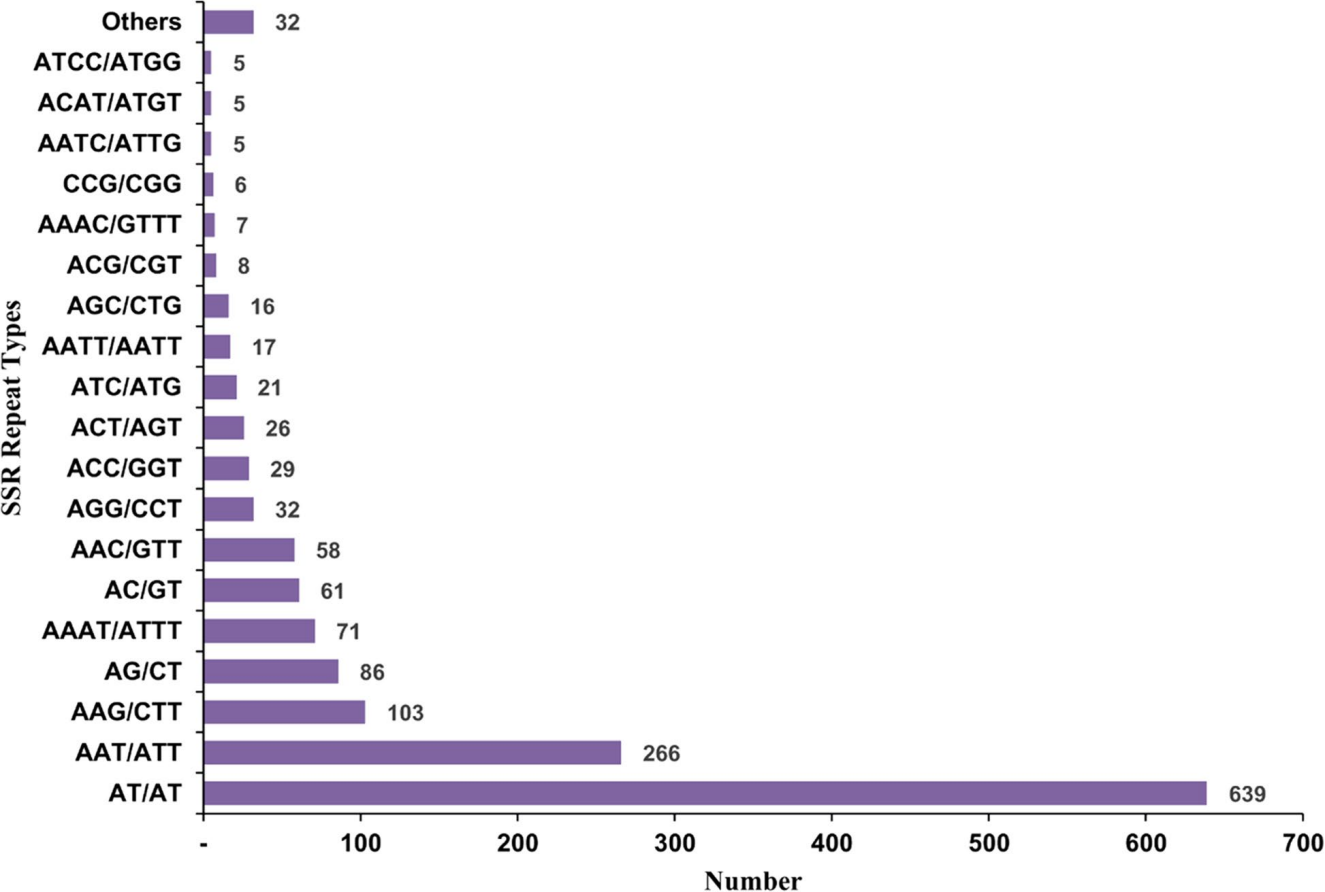
Numbers of SSR repeats of various types in *C. tripartitus* unigenes. The most common repeat types included the dinucleotide AT/AT and trinucleotide AAT/ATT

DNA elements were the most promiscuous repeating elements, with the hATCharlie and TcMar-Tigger elements being prominent in the *C. tripartitus* unigenes. LINEs such as LINE1, LINE2, and L3/CR1repeats occupied lengths of 372, 340, and 2,346 bp, respectively (Table 4). Among SINEs, only mammalian-wide interspersed repeats (MIR; 2 elements) were found among the unigenes, occupying 88 bp of length. The unigenes also contained simple repeats, low-complexity regions, and small RNAs. Among all repeating elements, simple repeats (8,082 elements) accounted for the greatest length (348,595 bp; 0.77% of all sequences). Next, we screened all unigenes for the presence of SSRs. A total of 1,493 SSR sequences were obtained from 1,212 unigenes, with 224 sequences containing more than 1 SSR. These SSRs were categorized based on the number of repeats as di-, tri-, tetra-, penta-, or hexanucleotide repeats (Table 5). Dinucleotide repeats were predominant (788 SSRs), followed by trinucleotide (565 SSRs) and tetranucleotide repeats (123 SSRs). Dinucleotide, trinucleotide, tetranucleotide and pentanucleotide repeats were present with a maximum of six, five, and four iterations, respectively. Further, under the SSR type classification (Fig. 9), we found that the dinucleotide repeat AT/AT (639 SSR) was predominant. Among trinucleotide repeats, AAT/ATT (266 SSRs) was the dominant SSR repeat type.

**Table 4:** RepeatMasker based analysis of repeating elements in *C. tripartitus* unigenes

		number of elements*	length occupied	percentage of sequence
SINEs:		4	201 bp	0.00%
	ALUs	0	0 bp	0.00%
	MIRs	2	88 bp	0.00%
LINEs:		55	6,960 bp	0.02%
	LINE1	7	372 bp	0.00%
	LINE2	5	340 bp	0.00%
	L3/CR1	29	2,346 bp	0.01%
LTR elements:		28	9,901 bp	0.01%
	ERVL	1	46 bp	0.00%
	ERVL-MaLRs	0	0 bp	0.00%
	ERV_class I	7	911 bp	0.00%
	ERV_class II	0	0 bp	0.00%
DNA elements:		320	80,947 bp	0.18%
	hAT-Charlie	135	34,337 bp	0.08%
	TcMar-Tigger	89	24,442 bp	0.05%
Unclassified:		1	86 bp	0.00%
Total interspersed repeats:			98,095 bp	0.22%
Small RNA:		15	6,941 bp	0.01%
Satellites:		0	0 bp	0.00%
Simple repeats:		8,082	348,595 bp	0.77%
Low complexity:		1,960	96,545 bp	0.21%

**Table 5:** Distribution and frequency of SSRs identified from all unigene sequences of *C. tripartitus* transcriptome

Repeats	4	5	6	7	8	9	10	11	12	13	14	15	16	17	18	19	20	21	Total
Di	0	0	383	215	70	51	36	14	10	0	1	0	2	1	4	0	0	1	788
Tri	0	473	81	6	5	0	0	0	0	0	0	0	0	0	0	0	0	0	565
Tetra	119	4	0	0	0	0	0	0	0	0	0	0	0	0	0	0	0	0	123
Penta	7	0	4	0	0	0	0	0	0	0	0	0	0	0	0	0	0	0	11
Hexa	2	1	0	0	0	0	2	1	0	0	0	0	0	0	0	0	0	0	6
Total	128	478	468	221	75	51	38	15	10	0	1	0	2	1	4	0	0	1	1493

### Candidate genes associated with the *C. tripartitus* immune defense system

Unigenes putatively involved in the physiological adaptation of *C. tripartitus* are listed in Table 6. Here, we emphasize that candidate genes such as aquaporin and heat shock protein 70 could be further characterized using functional genomics to elucidate the physiological adaptation of the species. An extensive summary of candidate genes assigned to immune defense categories such as ‘Pattern recognition receptor (PRR)’, ‘TLR signaling pathway’, ‘Adaptor proteins’, ‘MyD88-dependent pathway’, ‘Endogenous ligands’, ‘Immune effectors’, ‘Antimicrobial peptides’, ‘Cytokines and cytokine receptors’, ‘Apoptosis-related’, ‘Autophagy-related’ and ‘others’ are provided in Table S2.

**Table 6:** List of adaptation-related genes identified in *C. tripartitus* transcriptome

Candidate genes family		Unigene (no.)
**Full Name**	**Symbol**	
angiotensin I converting enzyme	ACE	1,146
adenylate cyclase activating polypeptide 1	ADCYAP1	1,887
angiotensinogen	AGT	1,923
AMP-activated protein kinase	AMPK	8,025
aquaporin 2	AQP2	5,211
basic helix-loop-helix family, member e40	BHLHE40	173
basic helix-loop-helix family, member e41	BHLHE41	265
chromosome 9 open reading frame 3	C9ORF3	19
collagen type I alpha 1	COL1A1	1,976
deiodinase, iodothyronine	DIO	721
endothelial PAS domain protein 1	EPAS1	134
glutamate ionotropic receptor delta type subunit 1	GRID1	94
glutamate ionotropic receptor NMDA type subunit 2B	GRIN2B	1,320
heat shock proteins 70	HSP70	4,443
insulin receptor substrate 1	IRS1	5,942
mitogen-activated protein kinase kinase kinase 15	MAP3K15	515
phospholipase A2 group XIIA	PLA2G12A	3
regulator of cell cycle	RGCC	363
somatolactin	SL	147
Solute Carrier	SLC	2
Stimulated by retinoic acid 6	STRA6	1,428
T-box 5	TBX5	2,962
Toll-like receptors4	TLR4	4,530

**Fig. 10:**
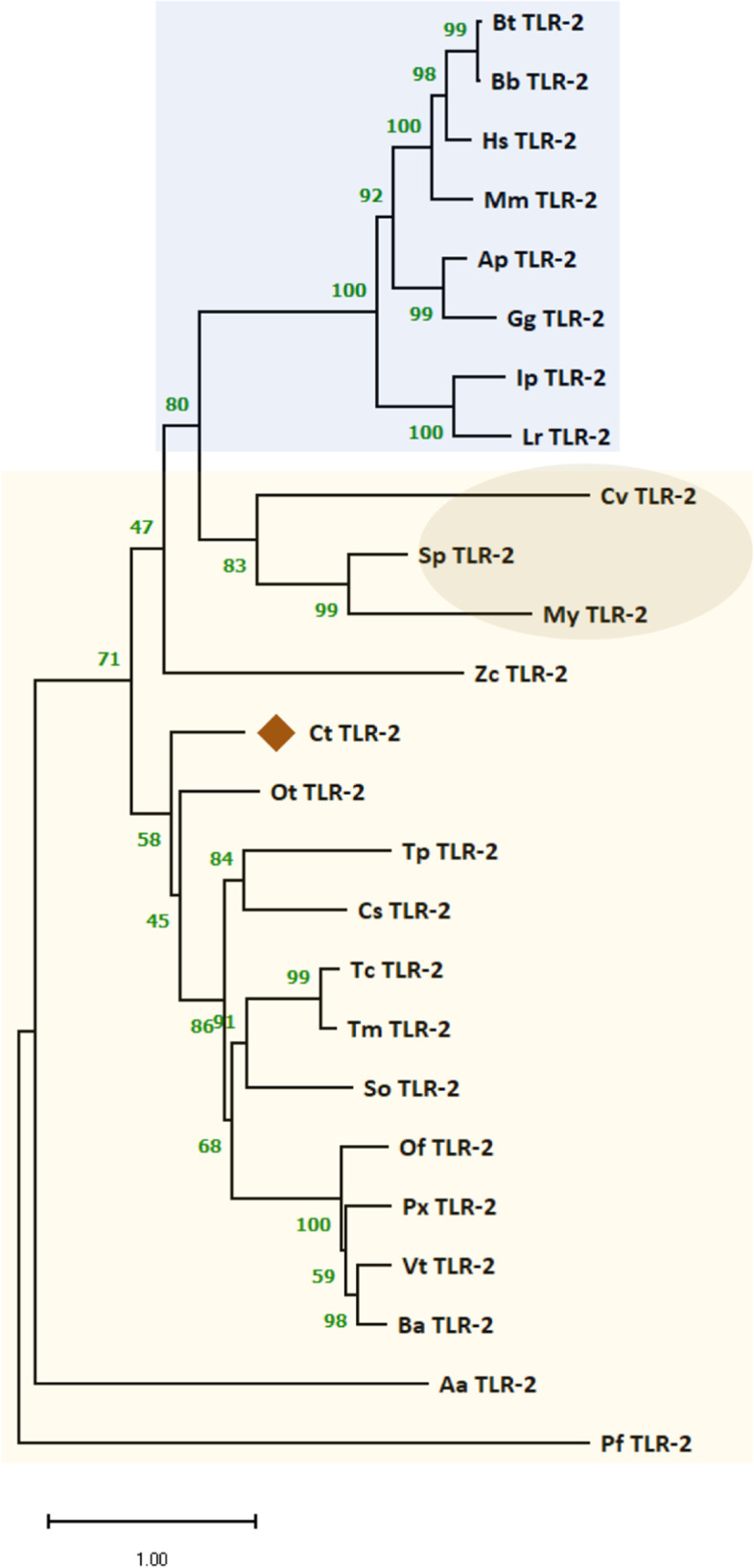
Phylogenetic analysis of Ct_TLR-2 with TLR-2 orthologs from representative invertebrate and vertebrate species. A bootstrap consensus tree (1,000 replicates) was constructed in MEGA 11.0 using the maximum-likelihood method. This analysis involved 25 amino acid sequences. All ambiguous positions were removed from each sequence pair (pairwise deletion option). The percentage of replicate trees in which the associated taxa clustered together is shown along each branch. The vertebrate and invertebrate taxa are shaded blue and yellow, respectively, and the molluscan cluster is circled.. GenBank accession numbers of the analyzed TLR-2 sequences are shown in parentheses. OtTLR-2, *Onthophagus taurus* TLR-2 (XP_022910857.1); TcTLR-2, *Tribolium castaneum* TLR-2 (XP_015837871.1); TmTLR-2, *Tribolium madens* TLR-2 (XP_044272570.1); SoTLR-2, *Sitophilus oryzae* TLR-2 (XP_030759691.1); ZcTLR-2, *Zeugodacus cucurbitae* TLR-2 (XP_011177598.1); OfTLR-2, *Ostrinia furnacalis* TLR-2 (XP_028171769.1); VtTLR-2, *Venessa tameamea* TLR-2 (XP_026487980.1); BaTLR-2, *Bicyclus anynana* TLR-2 (XP_023948157.1); PxTLR-2, *Papilio xuthus* TLR-2 (XP_013180232.1); PfTLR-2, *Polistes fuscatus* TLR-2 (XP_043489782.1); AaTLR-2, *Aricia agestis* TLR-2 (XP_041974768.1); TpTLR-2, *Thrips palmi* TLR-2 (XP_034250851.1); CsTLR-2, *Cryptotermes secundus* TLR-2 (XP_033607111.1); SpTLR-2, *Sepia pharaonis* TLR-2 (CAE1279087.1); CvTLR-2, *Crassostrea virginica* TLR-2 (XP_022314615.1); MyTLR-2, *Mizuhopecten yessoensis* TLR-2 (XP_021339985.1); MmTLR-2, *Mus musculus* TLR-2 (EDL15415.1); BtTLR-2, *Bos taurus* TLR-2 (ALL55248.1); ApTLR-2, *Anas platyrhynchos* TLR-2 (ATD82882.1); GgTLR-2, *Gallus gallus* TLR-2 (ATD82881.1); IpTLR-2, *Ictalurus punctatus* TLR-2 (AEI59663.1); LrTLR-2, *Labeo rohita* TLR-2 (ADQ74644.1); BbTLR-2, *Bubalus bubalis* TLR-2 (ANV28170.1); HsTLR-2, *Homo sapiens* TLR-2 (AAH33756.1)

We targeted the C-type lectin (Ct_CTL; unigene_11037), Peptidoglycan recognition Protein SC2-like (Ct_PGRP-SC2-like; unigene_12574), and Toll-like receptor-2 (Ct_TLR-2; unigene_22346) sequences screened from the PANM-DB-annotated unigene profile of *C. tripartitus* for detailed in silico analysis. This is because of their established role as PRRs related to innate immunity in insects. Ct_TLR2 is a 1,224-bp (with a predominance of A + T bases) ORF encoding a polypeptide of 407 amino acid residues (Figure S1). Aside from leucine-rich repeat (LRR) domains at the N-terminus, a conspicuous Toll-interleukin receptor (TIR) domain and type-I transmembrane region of 22 amino acids were identified in Ct_TLR2 protein sequence. Phylogenetic analysis demonstrated that Ct_TLR2 is related to other invertebrate TLR2 orthologs (Fig. 10). Ct_TLR2 shared maximum homology with TLR2 of the dung beetle *Onthophagus taurus* (Ot_TLR2). Vertebrate TLR2 protein sequences clustered separately from invertebrate TLR2 orthologs. The secondary structure prediction results indicate the presence of both β-strands and α-helices, with α-helices dominating the transmembrane region (Figure S2).

**Fig. 11:**
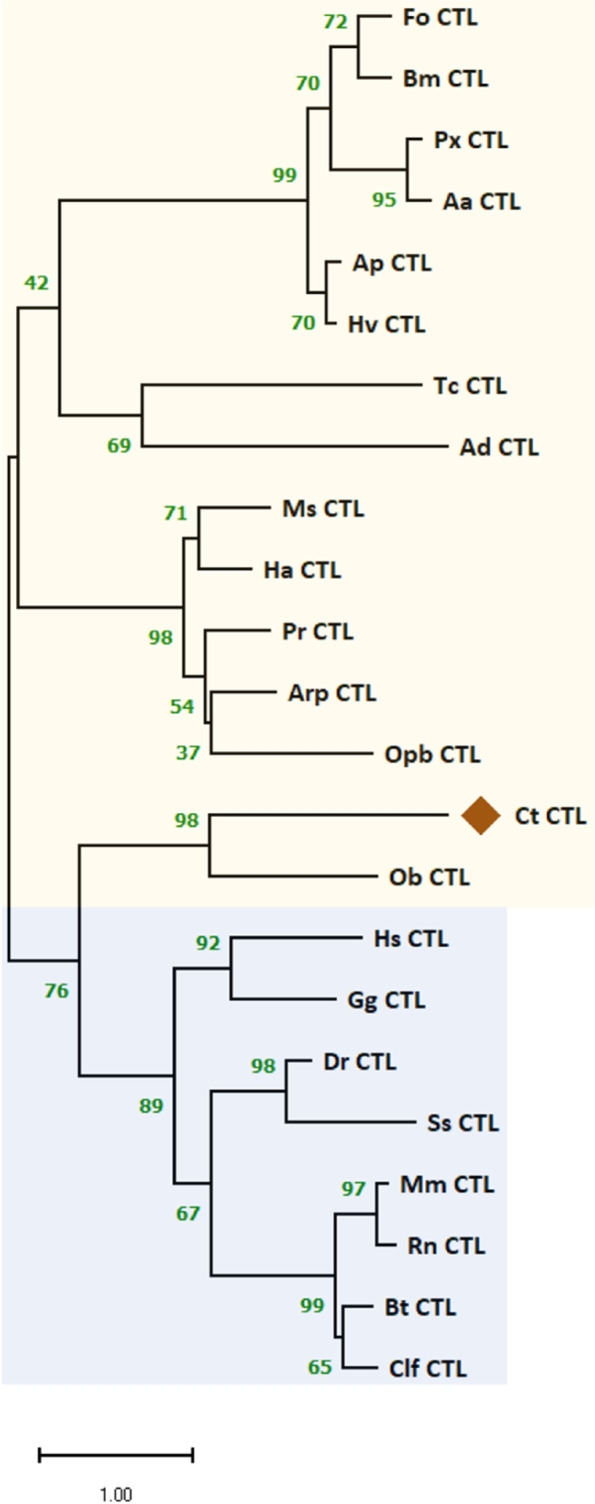
Phylogenetic analysis of Ct_CTL with the maximum-likelihood method using MEGA (version 11.0). The tree passed the bootstrap test of 1,000 replications and the resulting values are shown along the branches. This analysis involved 23 amino acid sequences. All ambiguous positions were removed from each sequence pair (pairwise deletion option). The vertebrate and invertebrate taxa are shaded in blue and yellow, respectively. GenBank accession numbers of the analyzed CTL sequences are shown in parentheses. FoCTL, *Frankliniella occidentalis* CTL (KAE8749903.1); BmCTL, *Bombyx mori* CTL (ABI79325.1); PxCTL, *Plutella xylostella* CTL (AFM52345.1); AaCTL, *Aedes aegypti* CTL (ABF18196.1); ApCTL, *Acyrthosiphon pisum* CTL (NP_001155798); HvCTL, *Homalodisca vitripennis* CTL (KAG8257308.1); TcCTL, *Tribolium castaneum* (XP_008193285); AdCTL, *Anopheles dirus* CTL (AFK83719.1); MsCTL, *Mythimna separata* CTL (BBC20960.1); HaCTL, *Helicoverpa armigera* CTL (ABF83203.1); PrCTL, *Pieris rapae* CTL (AEO52696.1); ArpCTL, *Antheraea pernyi* (AGN70857.1); OpbCTL, *Operophtera brumata* CTL (KOB78577.1); ObCTL, *Oryctes borbonicus* CTL (KRT82901.1); HsCTL, *Homo sapiens* CTL (AAG00514.1); GgCTL, *Gallus gallus* CTL (CAD61336.1); DrCTL, *Danio rerio* CTL (XP_005172687.1); SsCTL, *Salmo salar* CTL (ACI68944.1); MmCTL, *Mus musculus* CTL (AAD05125.1); RnCTL, *Rattus norvegicus* CTL (NP_001003707.1); BtCTL, *Bos taurus* CTL (NP_001180046.1); ClfCTL, *Canis lupus familiaris* CTL (XP_005637254.1)

The ORF of Ct_CTL was 1,071 bp and encoded a protein of 356 amino acid residues. Domain analysis showed a typical signal peptide sequence of 18 amino acid residues at the N-terminus with tandem CLECT [C-type lectin/carbohydrate-recognition domain (CRD)] domains (Figure S3). Ct_CTL clustered with CTL homologs from representative beetle species, and most closely with that of the scarab beetle, *O. borbonicus* CTL (Ob_CTL). Other clusters were associated with mammalian and insect CTLs (Fig. 11). The secondary structure of Ct_CTL contained three predicted α-helical regions at the N-terminus and three α-helical regions at the CLECT (C-type lectin) domain interspersed with short β-sheet elements (Figure S4). Although two unigenes represented CTLs in the annotation results (Table S2), both had 100% identity in the BLASTp analysis results.

**Fig. 12:**
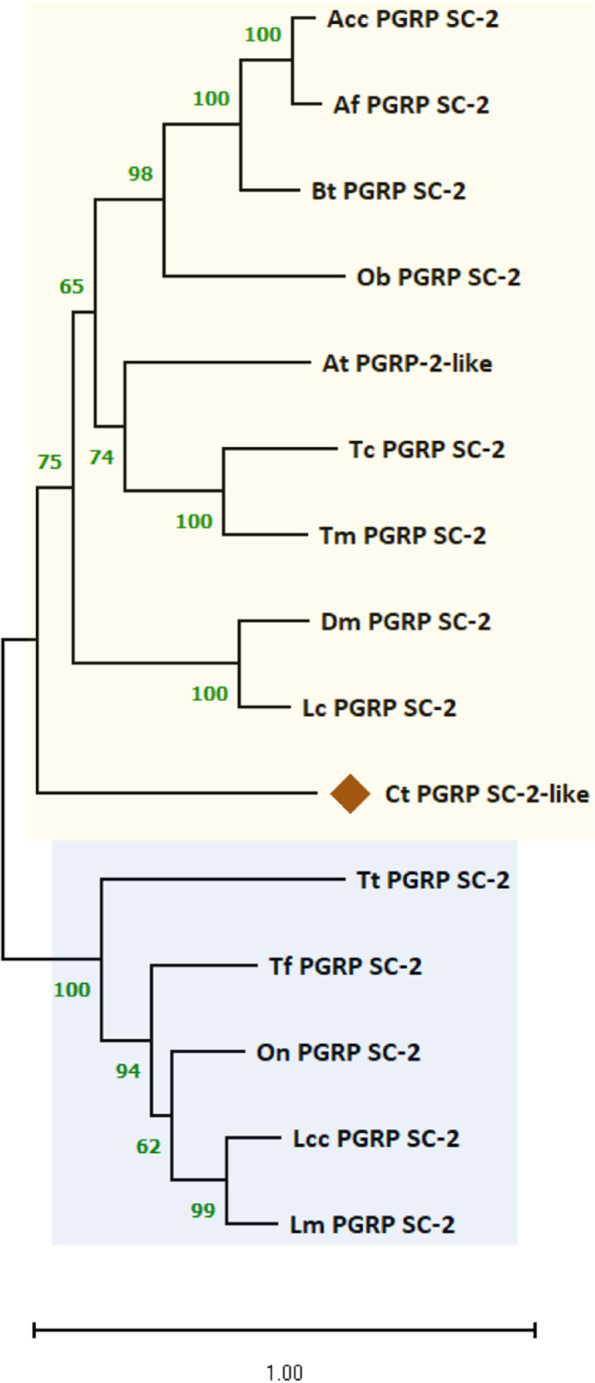
Phylogenetic analysis of Ct_PGRP_SC-2-like along with other representative PGRP amino acid sequences from vertebrates and invertebrates. Molecular phylogeny was inferred using the maximum-likelihood method in MEGA software (version 11.0). This analysis involved 15 amino acid sequences. The tree was bootstrapped (1,000 replications) and the values are shown along the branches. All ambiguous positions were removed from each sequence pair (pairwise deletion option). The vertebrate and invertebrate taxa are shaded in blue and yellow, respectively. GenBank accession numbers of the analyzed sequences are shown in parentheses. Acc PGRP SC-2, *Apis cerana cerana* PGRP SC-2 (PBC31638.1); Af PGRP SC-2, *Apis florea* PGRP SC-2 (XP_003694493.1); Bt PGRP SC-2, *Bombus terrestris* PGRP SC-2 (XP_012170795.1); Ob PGRP SC-2, *Ooceraea biroi* PGRP SC-2 (EZA50446.1); At PGRP-2-like, *Aethina tumida* PGRP-2-like (XP_019877658.1); Tc PGRP SC-2, *Tribolium castaneum* PGRP SC-2 (XP_008193407.1); Tm PGRP SC-2, *Tenebrio molitor* PGRP SC-2 (BAJ23047.1); Dm PGRP SC-2, *Drosophila melanogaster* PGRP SC-2 (CAD89178.1); Lc PGRP SC-2, *Lucilia cuprina* PGRP SC-2 (KNC21676.1); Tt PGRP SC-2, *Triplophysa tibetana* PGRP SC-2 (KAA0714663.1); Tf PGRP SC-2, *Takifugu flavidus* PGRP SC-2 (TWW77559.1); On PGRP SC-2, *Oreochromis niloticus* PGRP SC-2 (ALM04192.1); Lcc PGRP SC-2, *Larimichthys crocea* PGRP SC-2 (KAE8295497.1); Lm PGRP SC-2, *Lateolabrax maculatus* PGRP SC-2 (QQR13803.1)

Further, the predicted PGRP-SC homolog (Ct_PGRP_SC-2-like) showed a full-length ORF of 567 nucleotides that translates to a protein of 188 amino acid residues (Figure S5). Domain analysis identified a typical signal peptide sequence of 19 amino acid residues at the N-terminus, and overlapping PGRP amidase activity (N-acetylmuramoyl-L-alanine amidase) domains from Pro-22 to Gly-163 (PGRP domain) and Gly-32 to Gly-169 (amidase_2 domain). On the evolutionary tree, Ct_PGRP_SC-2-like is not closely clustered with any orthologs but groups with the invertebrate PGRP_SC-2 cluster, while vertebrate PGRP_SC-2 orthologs form a separate cluster (Fig. 12). The predicted secondary structure of Ct_PGRP_SC2-like contained six α-helices and six β-strands (Figure S6).

### Candidate genes related to growth and muscle development

Whole-body transcriptome characterization of *C. tripartitus* allowed for the identification of unigenes putatively related to the somatotropic axis and muscle growth. Candidate unigenes related to the somatotropic axis included *insulin-related peptide*, *epidermal growth factor receptor*, *adenosine deaminase* and other transcription factors. We further identified unigenes, including *actin* and *tropomyosin,* related to the muscle growth, as well as unigenes related to overall growth and development such as *chitinase*, *collagen*, *apolipophorins*, and *calcitonin* (Table S3).

### Candidate genes related to sex determination and reproduction

Genetic factors putatively related to sex determination and differentiation were also identified among unigenes of *C. tripartitus*, including the transcription factor *Sox-2*, protein *MAB-21*, and *GATA zinc finger domain*. Putative unigenes for the sex-determining protein *fem-1* (feminization-1) were abundant in the transcriptome. Among reproduction-related unigenes, we identified genes associated with *spermatogenesis-associated protein, sperm surface protein, spermidine synthase, spermine oxidase, testis expressed sequences, vitellogenin*, and kinases (Table S4).

## Discussion

Mapping of regulatory transcripts using the transcriptome characterization approach has been successfully applied to elucidate the fitness traits necessary for ecological sustenance of non-model invertebrate species including insects [1, 2, 28]. Most such studies have employed Illumina sequencing platform and Trinity-based de novo assembly as it provides high-quality sequences for functional annotation and microsatellite discovery. This was the first study to characterize the transcriptome of the endangered paracoprid beetle *C. tripartitus,* which was screened for putative regulatory transcripts involved in immunity, growth, and reproduction, while also sufficiently addressing the need for microsatellite markers for use in population genetic studies. Notably, the highly polymorphic and codominant SSR markers obtained from transcriptome sequencing are highly transferable and can be used for diversity studies of related species, including other genera of the same family [29, 30]. The development of reference transcriptome provides an insight into fitness traits of the beetle and will contribute towards understanding the sustainability of paracoprid beetles in the wild and their maintanence of ecosystem health.

The de novo assembled unigenes obtained in this study showed higher N50 and mean length compared to transcriptome assemblies of the social caterpillar *Drepana arcuata* [31], coffee berry borer, *Hypothenemus hampei* [32], and Lycaenidae butterflies *S. takanosis* and *P. superans* [1]. While annotating the unigenes, the PANM-DB was found to be more reliable in terms of annotation hits and time of annotation [19]. The species distribution of the top matches to *C. tripartitus* unigenes showed a bias towards completely sequenced beetle genomes, such as *T. castaneum* and *O. borbonicus*, and other non-model insects.

The IPS-based conserved domain search identified C2H2-like zinc-finger, protein kinase, immunoglobulin-like fold, carboxylesterase type B, zinc finger (RING-type), and reverse transcriptase as being among the most abundant domains in the unigenes. Such domains were also widely distributed in transcriptome-derived unigenes of *H. hampei* [32], Asian giant hornet *V. mandarinia* [18], Nymphalid butterfly *Fabriciana nerippe* [2], and Mexican bean weevil *Zabrotes subfasciatus* [33]. C2H2-like zinc-finger domains are among the most abundant protein domains belonging to the family of transcription factors that regulate gene expression in complex eukaryotes. C2H2 proteins with three C2H2 domains have been less thoroughly studied [34, 35]. The catalytic domain features of protein kinases function intracellularly in phosphorylation and promote numerous signaling cascades related to metabolic, cellular and immune processes. Immunoglobulin-like fold domains provide interacting surfaces for the binding of other proteins via their β-sheets [36]. Carboxylesterases containing the carboxylesterase domain are responsible for various physiological functions related to insect development and behavior, and are broadly distributed among insects such as *Lucilia cuprina*, *Musca domestica*, and *Anopheles gambiae* [37]. Further, our GO-based functional annotations showed an over-representation of sub-functional categories such as cellular process, metabolic process, and single-organism process (within the Biological process category); binding and catalytic activity (within the Molecular function category); and cell, cell part, and membrane (within the Cellular component category) as reported in transcriptome annotations of other beetles [38–40]. As known, GO annotations are based on GO evidence code distributions. Most of the GO evidence codes refer to ‘electronic’ annotations that are not experimentally defined and hence the functional directions of unigenes can only be predictive. Insights into the biochemical pathways were provided by the KEGG analysis, wherein a significant number of unigenes encoding enzymes categorized under metabolic pathways [41]. The distribution of KEGG- annotated unigenes in well-represented metabolic pathways has been suggested previously for the Sakhalin pine sawyer *Monochamus saltuarius* [42] and other lepidopteran insects [43, 44]. Furthermore, the distribution of *C. tripartitus* unigenes to the KOG functional category ‘Signal transduction’ is significant, as most immune processes in insects have conserved components under various signaling cascades determining host resistance or susceptibility to pathogenic infections. Similar KOG classification results have been obtained for the seed beetles, *Callosobruchus maculatus*, Japanese pine sawyer beetle *Monochamus alternatus*, and pine shoot beetle *Tomicus yunnanensis* [38, 39, 45].

We further analyzed the repeating elements and SSRs in de novo assembled unigenes of *C. tripartitus*. Repeating elements such as retroelements play significant roles in adaptive processes and contribute to phenotypic plasticity [46, 47]. Transposed elements such as SINEs, LINEs, and LTR elements inserted into exons can elongate the untranslated region (UTR) and contribute to genomic expansion, genomic rearrangement, and genetic diversity [48]. Further, the discovery of polymorphic microsatellite markers has gained importance because they are ideal molecular marker system for investigating genetic diversity [49], and can reveal genes directly related to physiology and adaptation [50]. The SSR and single-nucleotide polymorphism (SNP) markers screened from transcriptomic resources of non-model species, including insects, have been widely applied in conservation genomics. For example, in populations of insect pest *Rhopalosiphum padi*, 60 randomly selected microsatellites (out of 7,936 potential microsatellites) were amplified using specific primer pairs to identify 14 polymorphic loci demonstrating successful utilization of microsatellites to elucidate genetic heterogeneity among *R. padi* populations and other closely related aphid species [30]. Further, consistent with our results, trinucleotide repeats were abundantly distributed among *R. padi* SSRs, as were mononucleotide repeats [30]. Mononucleotide repeats were not considered in the present study due to the propensity for homopolymer formation during Illumina sequencing. Dinucleotide and trinucleotide repeats were also reported as the most abundant SSRs in the transcriptome of sawfly *Dolerus aeneus*, stick insect *Timema cristinae*, and oriental fruit fly *Bactrocera dorsalis* [51–53]. Further, dinucleotides were most abundant among unigenes of the red palm weevil *Rhynchophorus ferrugineus* with AT and TA accounting for more than half of all dinucleotide motifs [54].

Innate immunity is essential for the adaptability of insect species to varying environments. It drives physiological plasticity in invertebrates, including insects [55]. Innate immunity has been studied at the molecular level in beetles, especially *T. castaneum* and *Tenebrio molitor* unraveling the mysteries of host–pathogen interactions. *T. molitor* transcriptome data have revealed the critical components of Toll, IMD, JAK-STAT, and autophagy-related signaling in the context of pathogenic infections [56–59]. Reflecting the physiological plasticity to various immune elicitors, studies on the antimicrobial innate immune response of *T. molitor* via the transcriptional regulation of AMPs have been richly insightful [60, 61]. Innate immune signaling components, including PRRs, membrane proteins, intracellular proteins such as kinases, and NF-kappaB molecules have been functionally characterized after initial screening of the transcriptome [60, 62, 63]. In this study, we screened the conserved components of innate immunity from *C. tripartitus* transcriptome that could provide clues about the successful habitation of this species to microbially-sensitive environments, and the mechanism of pathogen evasion. In the context of innate immunity, PRRs such as lectins, TLRs, and glucan- and lipopolysaccharide- binding molecules in the extracellular, membrane, and intracellular environments can establish direct interactions with PAMPs and modulate signaling cascades related to innate immunity [64]. The repertoire of such PRRs is diverse in simple animals, likely due to gene duplication, and this large repertoire supports multiple binding affinities to PAMPs and complex signaling cascades. We preliminarily characterized the PRRs obtained from the *C. tripartitus* transcriptome, including TLR-2, PGRP-SC2-like, and CTL, in an explicitly phylogenetic context.

TLRs are by far the most comprehensively studied class of proteins in relation to the innate immune system. TLRs are single, membrane-spanning, non-catalytic receptors that recognize structurally conserved molecules derived from microbes. These proteins regulate molecular traffic between the plasma membrane and endosome [65]. TLRs contain the consensus ‘Leu-x-x-Leu-x-Leu-x-x-Arg’ protein-protein interaction module or the LRR domain [66], which are conserved across species and specifically enriched in plants [67], invertebrates [68], and cephalochordates [69]. The large repertoire of TLRs in animals is attributed to their role in the recognition of pathogens, leading to the development of diverse innate immune signaling cascades. The number and amino acid framework of the LRR motifs in TLRs lend credence to the binding stability of TLRs to PAMPs [66]. TLR proteins from the *C. tripartitus* transcriptome (TLR-2, TLR-6, and TLR-7) may act as membrane receptors to drive the MyD88-dependent pathway of the Toll cascade with assistance from intracellular components. Consistent with our results, core genes of the TLR pathway (*TLRs, MyD88,* and kinases) have also been identified in the transcriptome of gypsy moth, *Lymantria dispar* [70].

CTLs are an important type of PRR playing diverse physiological roles in animals, including humans and insects [71, 72]. CTLs are characterized by their capacity to possess one or more CRDs (also known as CTL domains) [73, 74]. Insect CTLs facilitate pattern recognition, agglutination, encapsulation, melanization, prophenoloxidase activation, and maintanence of gut microbiome homeostasis [75]. We screened CTL homologs in the *C. tripartitus* transcriptome. Ct_CTL encodes a protein of 356 amino acid residues with tandem CTL domains, while *Bombyx mori* CTL-S2 encodes a protein of 221 amino acids [76] and *Plutella xylostella* CTL encodes a protein of 322 amino acids with a dual CTL domain [77]. Further, while the CTLs of most insects show species-specific gene expansion, *B. mori* CTLs are widely distributed among the clades of CTLs of lepidopteran insects [78]. Moreover, we found that Ct_CTL clusters with the CTL of another beetle, *O. borbonicus,* implying close sequence identity of CTL homologs among coleopteran insects. PGRPs specifically bind to peptidoglycan present in the cell surface of bacteria and are classified into PGRP-L (long-form) and PGRP-S (short-form) types, which are widespread across invertebrate and vertebrate phyla. PGRPs participate in lytic attack of the bacterial cell wall [79] and promote cellular phagocytosis [80]. PGRPs (both L- and S-forms) have been identified in insects such as *Drosophila melanogaster* [81], *A. gambiae* [82], *B. mori* [83], *Nilaparvata lugens* [84], and *Sogatella furcifera* [85]. Both L- and S-type PGRPs were identified in the transcriptome of *C. tripartitus*. The S-form of PGRP (PGRP_SC2) screened from the *C. tripartitus* transcriptome contains the characteristic overlapping PGRP and amidase domains, and a 19-amino acid signal peptide sequence. Insect S-form PGRPs all contain signal peptide sequences, with *Drosophila* PGRP_SC2 also showing amidase activity [86]. These PGRPs also contribute to downregulation of the immune deficiency (IMD) pathway in the fat body of insects following systemic bacterial infection. PGRP_SC2, PGRP_SC1a and PGRP_LB are catalytic PGRPs; and in contrast to non-catalytic PGRPs (PGRP_LC, -LE, -SA, and –SD), they have a cysteine residue in the active site for peptidoglycan cleavage [87]. Similarly, a secreted PGRP_SC2 homolog from the genome of the mosquito *Aedes aegypti* has been predicted to function as a negative regulator of immune responses [88].

Genes associated with growth and development are critical to the success of insects in the wild, and for translocation to new habitats. As *C. tripartitus* is designated an endangered species in Korea, permission was granted to collect only three individuals. This prevented developmental stage-specific transcriptome analysis, which could elucidate the growth and development attributes of *C. tripartitus*. Moreover, genome-wide analysis of developmental stage-specific transcriptome data is crucial to the development of novel control approaches for insect pests, although this goal is far removed from the objectives of this study. Developmental transcriptome analysis has been conducted for an endangered Korean butterfly, *Parnassius bremeri,* to clarify its population genetics and inform conservation measures [89]. The cataloging of transcripts (such as actin, myosin, and tropomyosin) with key functions in growth and muscle development has been achieved in studies of the ecological dynamics of non-model species, and has provided molecular resources for future breeding programs [90].

Genes involved in the differentiation of gonadal structures, such as the ovary and testis, are sex-determination genes. The development of gonadal structures might be indirectly influenced by environmental factors such as light, temperature, nutritional conditions, and the reproductive physiology of the species via genetic regulation. In this context, discussion of successful reproduction strategies and genetic factors governing sex determination is pertinent. Gonadal transcriptome analysis of insect species led to the identification of candidate genes involved in sex-determination/differentiation and reproduction, thereby providing a scientific basis for exploring sex-related economic traits associated with disease resistance and the overall health of the organism [91]. In the transcriptome of *R. ferrugineus,* 25 genes were annotated as relevant to reproduction, including five vitellogenin transcripts; however, only a single vitellogenin gene was expressed [54], consistent with reports of a single vitellogenin gene in other coleopteran species such as *T. molitor* [92]*, Anthonomus grandis* [93]*, Octodonta nipae* [94]*,* and *Colaphellus bowringi* [95]. Those transcripts (designated *vitellogenin-1, -2,* and *-6-like*) were also found in the transcriptome of *C. tripartitus,* implying a substantial contribution to the reproductive success of insects. Vitellogenin significantly contributes to ovarian development in insects through lipid accumulation in the ovaries [96].

## Conclusions

This study provides a reference transcriptome for the dung beetle species *C. tripartitus*, which is classified as an endangered species in South Korea. The novel characterization of molecular resources (immunity, growth, and reproduction-related transcripts) from this species will be useful for benchmarking fitness traits in *C. tripartitus*, which may increase its adaptation potential in the wild. The 25,106 non-redundant unigenes identified here enable gene discovery and functional genomics in *Copris* species, and improve understanding of beetle and insect immunity. We assessed the ORF and putative protein characteristics of PRRs, such as TLR-2, CTL, and PGRP_SC-2-like, using in silico methods. Further insights into the innate immune signaling cascades of beetles and other insects were obtained through exploration of the components of the MyD88-dependent pathway, antimicrobial peptides, autophagy, and apoptosis pathways. A significant number of putative sex-determination/reproduction and growth-related transcripts were identified in the paracoprid dung beetle transcriptome, which sheds light on the habit and habitat requirements of this species.

## Supplementary Information

**Additional file 1**: **Figure S1.** The full-length nucleotide sequence for C*. tripartitus* Toll-like receptor-2 (Ct_TLR-2). **Figure S2.** Secondary structure prediction of Ct_TLR2 using PSI-PRED (version 4.0). **Figure S3.** The full-length nucleotide sequence for *C. tripartitus* CTL (C-type Lectin; Ct_CTL). **Figure S4.** Secondary structure prediction of Ct_CTL using PSI-PRED (version 4.0). **Figure S5.** The full-length nucleotide sequence for *C. tripartitus* Peptidoglycan Recognition Protein SC-2-like (Ct_PGRP_SC-2-like). **Figure S6.** Secondary structure prediction of Ct_PGRP_SC-2-like using PSI-PRED (version 4.0).

**Additional file 2:** **Table S1.** Preprocessing of raw reads obtained from *C. tripartitus* using Illumina next-generation sequencer. **Table S2.** Classification of *C. tripartitus *Candidate genes to the innate immune signaling process. **Table S3.** Genes of interest related to growth in the dung beetle, *C. tripartitus*. **Table S4.** Candidate Sex-Determination and Reproduction related genes from *C. *tripartitus unigenes.

## Data Availability

The datasets generated and analyzed during the current study are available from the Sequence Read Archive (SRA) of the National Center for Biotechnology Information (NCBI) under accession number PRJNA559824.

## Abbreviations

AMPs: Antimicrobial peptides
ORF: Open reading frame
PANM-DB: Protostome database
SSR: Simple sequence repeats
PRRs: Pathogen recognition receptors
CTL: C-type lectins
PGRP: Peptidoglycan recognition proteins
TLR: Toll-like receptors
PAMP: Pathogen- associated molecular patterns
IMD: Immune deficiency
RIN: RNA integrity number
NGS: Next-generation sequencing
NCBI: National center for biotechnology information
KOG: Clusters of orthologous groups
GO: Gene ontology
KEGG: Kyoto encyclopedia of genes and genomes
MEGA: Molecular evolutionary genetics analysis
MISA: MicroSatellite
LRR: Leucine-rich repeats
JAK-STAT: Janus kinase-signal transducer and activator of transcription
SINE: Short interspersed nuclear elements
LINE: Long interspersed nuclear elements
LTR: Long-terminal repeats
IPS: InterProScan
SNP: Single nucleotide polymorphism
TIR: Toll-interleukin receptor
CRD: Carbohydrate-recognition domain
UTR: Untranslated region
