# Supplementary material for: Transcriptome analysis of the endangered dung beetle Copris tripartitus (Coleoptera: Scarabaeidae) and characterization of genes associated to immunity, growth, and reproduction
Source: BMC Genomics. 2023 Mar 2;24:94. doi: 10.1186/s12864-023-09122-w (PMC9979532; doi:10.1186/s12864-023-09122-w)

**Supplementary Figures**

**Figure S1.** The full-length nucleotide sequence for C*. tripartitus* Toll-like receptor-2 (Ct_TLR-2). The predicted ORF sequences and the translated protein sequences are denoted as three-letter code and one-letter code, respectively. The stop codon is shown by an asterisk (*). The conserved Toll/Interleukin-1 receptor (TIR) domain is boxed. The Leucine-rich repeat regions (LRRs) are highlighted in red font. The transmembrane region is underlined. The N-Glycosylation and Phosphorylation sites are represented in bold and Italics font, respectively.

**Figure S2.** Secondary structure prediction of Ct_TLR2 using PSI-PRED (version 4.0). Cylinders in pink represent alpha helices, yellow bars represent beta strands and black lines represent coils.

**Figure S3.** The full-length nucleotide sequence for *C. tripartitus* CTL (C-type Lectin; Ct_CTL). The predicted ORF with the deduced amino acid sequences are denoted as three-letter code and one-letter code, respectively. The tandem CLECT [(C-type lectin/CTL) / carbohydrate-recognition domain (CRD)] domain of CTL protein is boxed. Serine, Threonine, and Tyrosine phosphorylation sites are indicated in italics font. The N-Glycosylation (sugar binding residues) sites are represented in bold. The signal peptide of 18 amino acid residues is underlined.

**Figure S4.** Secondary structure prediction of Ct_CTL using PSI-PRED (version 4.0). Cylinders in pink represent alpha helices, yellow bars represent beta strands and black lines represent coils.

**Figure S5.** The full-length nucleotide sequence for *C. tripartitus* Peptidoglycan Recognition Protein SC-2-like (Ct_PGRP_SC-2-like). The conserved PGRP and overlapping amidase_2 domains are boxed and represented in purple font, respectively. The signal peptide of 19 amino acid residues is underlined. The N-glycosylation and phosphorylation sites are shown in bold and italics font, respectively.

**Figure S6.** Secondary structure prediction of Ct_PGRP_SC-2-like using PSI-PRED (version 4.0). Cylinders in pink represent alpha helices, yellow bars represent beta strands and black lines represent coils.

**Figure S1**


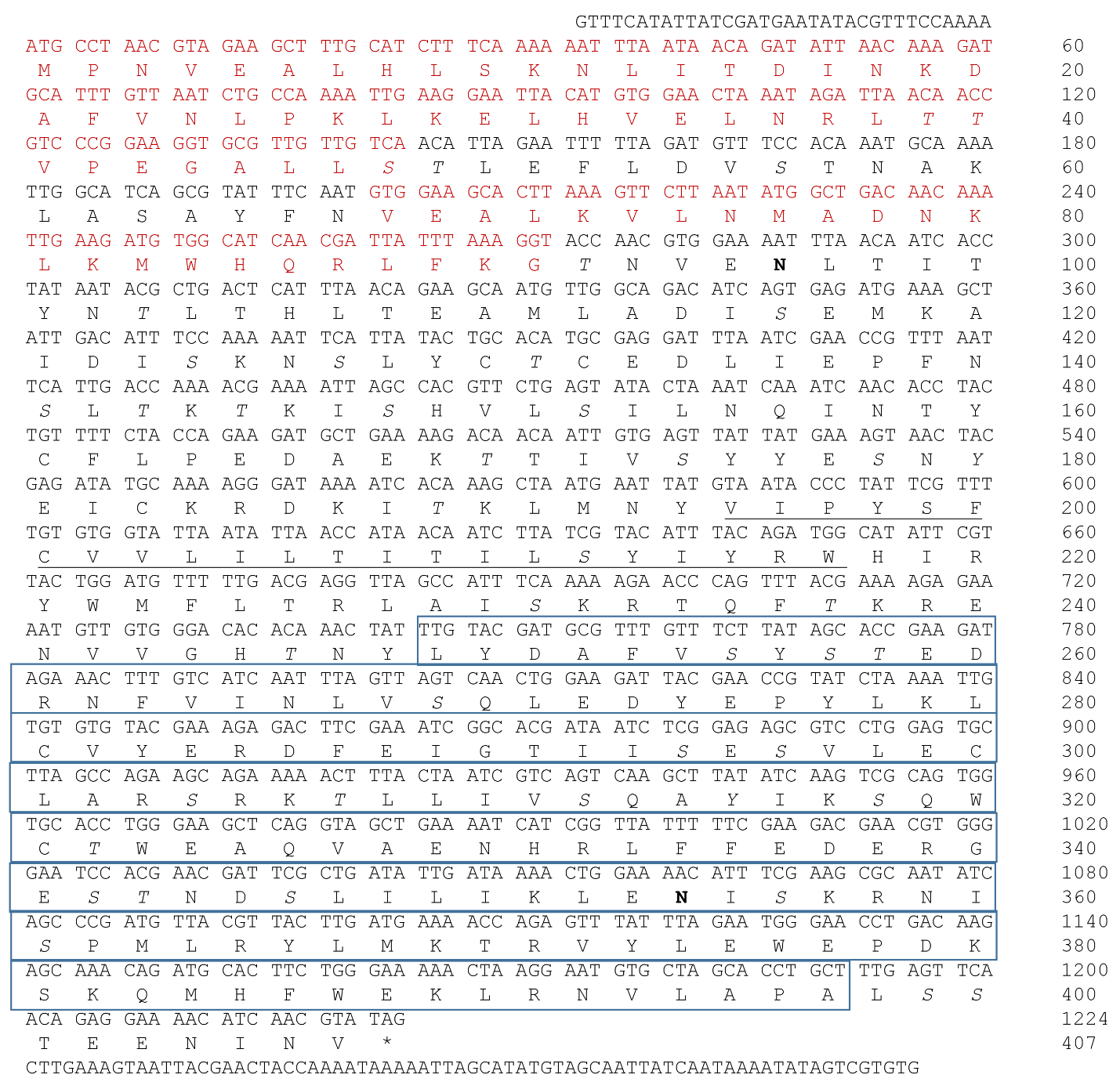


**Figure S2**


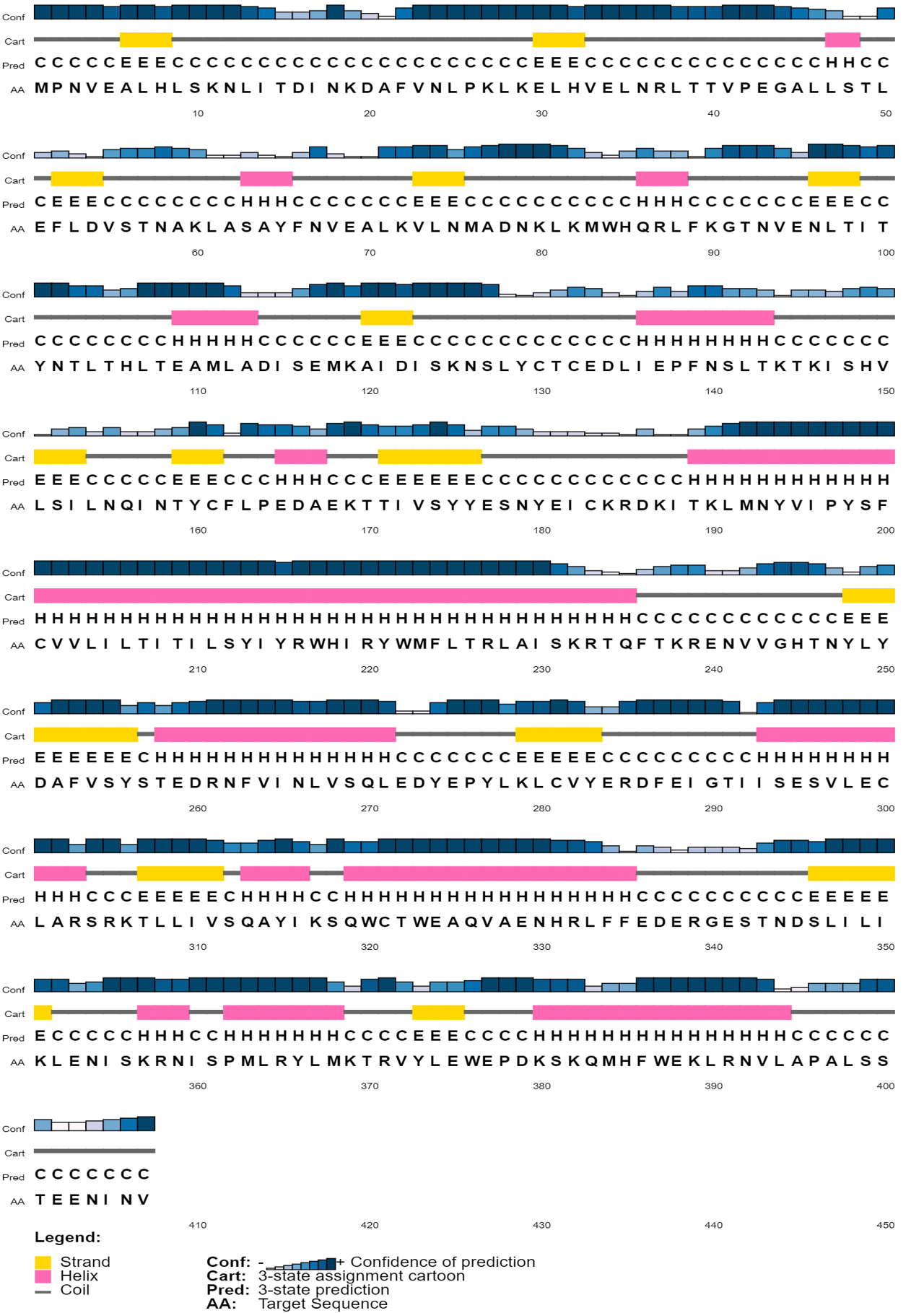


**Figure S3**


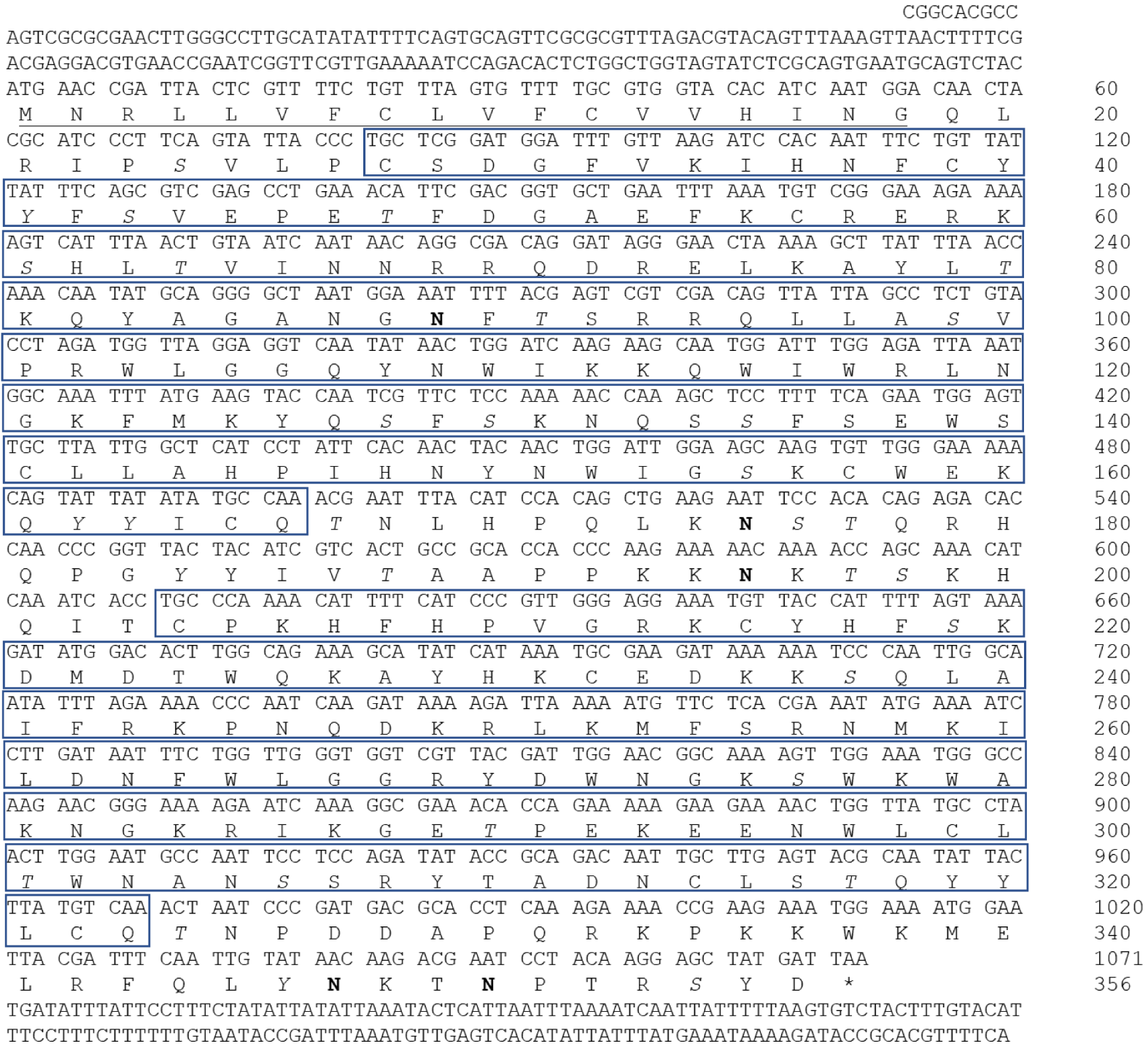


**Figure S4**


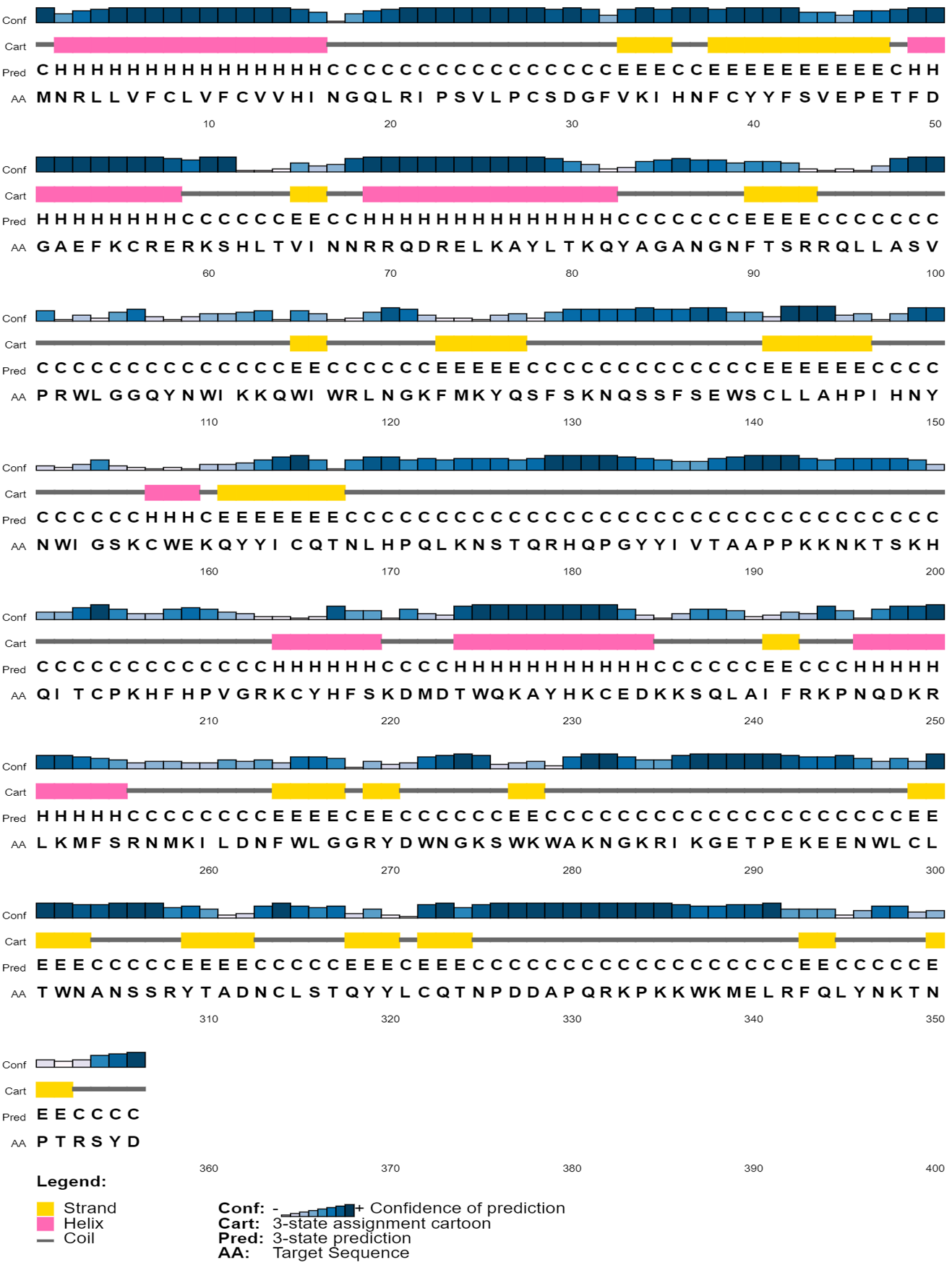


**Figure S5**


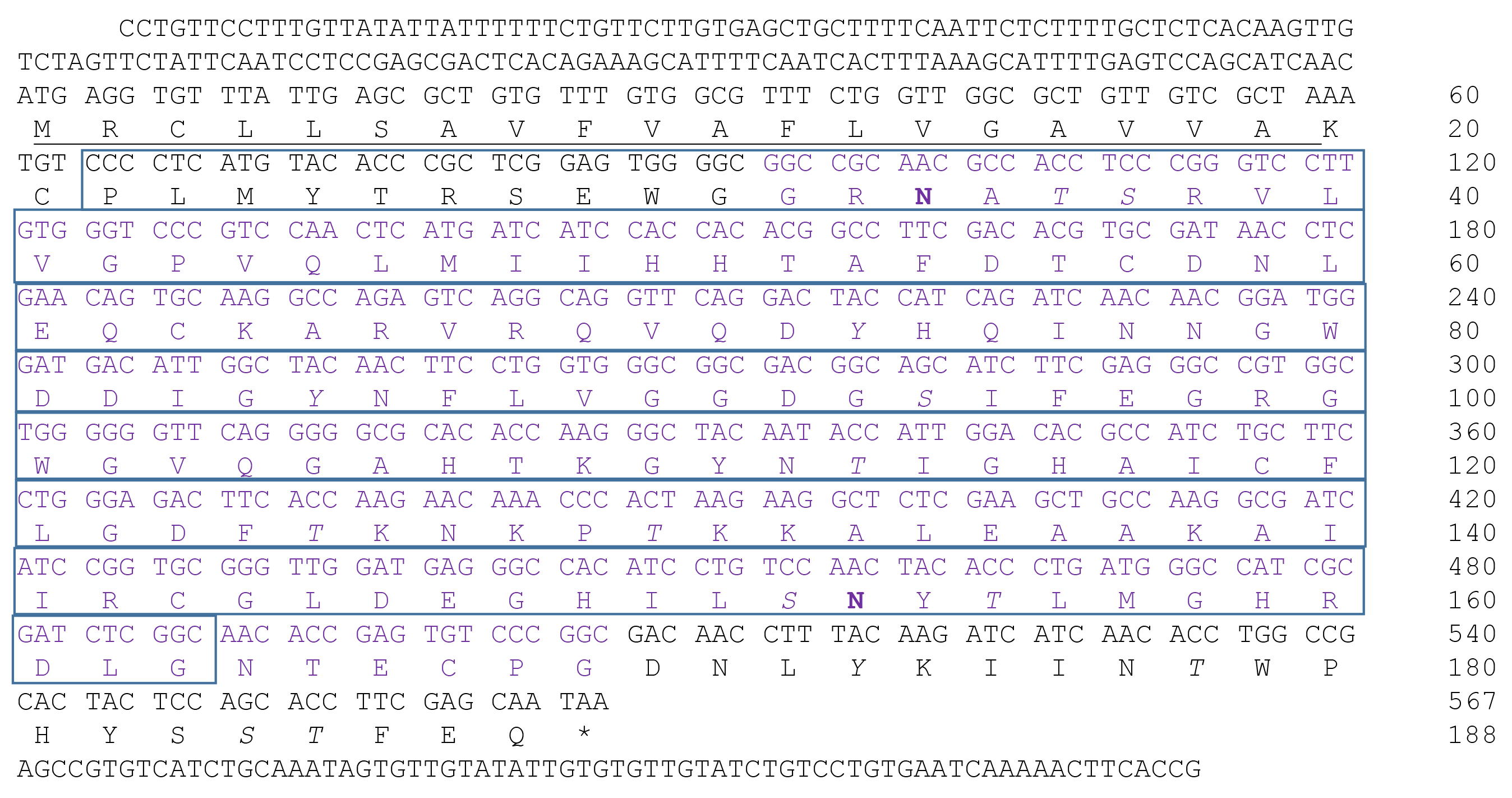


**Figure S6**


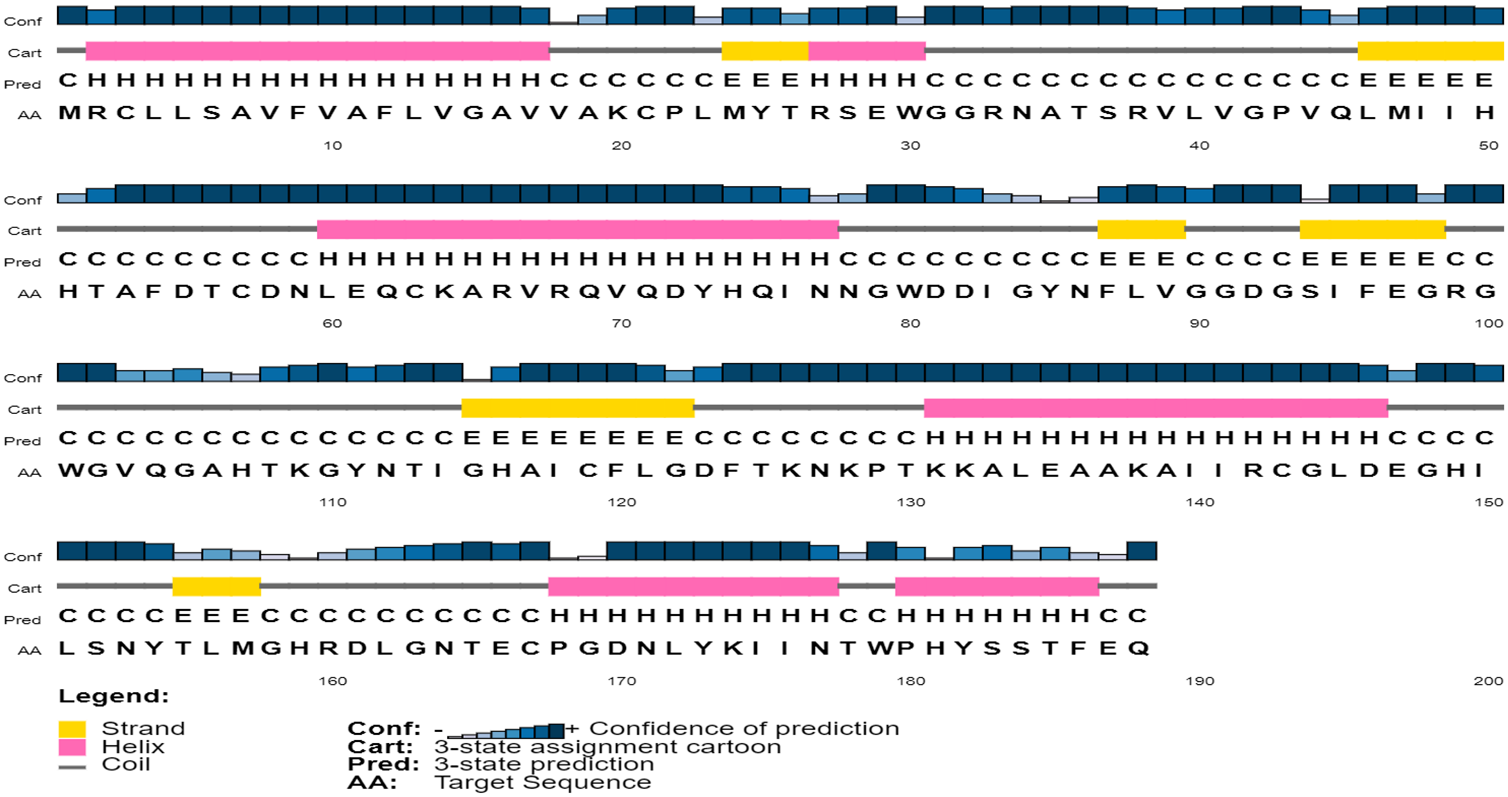

Supplement: Supplementary file 1 — Additional file 1: Figure S1. The full-length nucleotide sequence for C. tripartitus Toll-like receptor-2 (Ct_TLR-2). Figure S2. Secondary structure prediction of Ct_TLR2 using PSI-PRED (version 4.0). Figure S3. The full-length nucleotide sequence for C. tripartitus CTL (C-type Lectin; Ct_CTL). Figure S4. Secondary structure prediction of Ct_CTL using PSI-PRED (version 4.0). Figure S5. The full-length nucleotide sequence for C. tripartitus Peptidoglycan Recognition Protein SC-2-like (Ct_PGRP_SC-2-like). Figure S6. Secondary structure prediction of Ct_PGRP_SC-2-like using PSI-PRED (version 4.0). [file 12864_2023_9122_MOESM1_ESM.docx]
