# Supplementary material for: Transcriptome analysis of the endangered dung beetle Copris tripartitus (Coleoptera: Scarabaeidae) and characterization of genes associated to immunity, growth, and reproduction
Source: BMC Genomics. 2023 Mar 2;24:94. doi: 10.1186/s12864-023-09122-w (PMC9979532; doi:10.1186/s12864-023-09122-w)
Supplement: Supplementary file 2 — Additional file 2: Table S1. Preprocessing of raw reads obtained from C. tripartitus using Illumina next-generation sequencer. Table S2. Classification of C. tripartitus Candidate genes to the innate immune signaling process. Table S3. Genes of interest related to growth in the dung beetle, C. tripartitus. Table S4. Candidate Sex-Determination and Reproduction related genes from C. tripartitus unigenes. [file 12864_2023_9122_MOESM2_ESM.docx]

**Table S1: Preprocessing of raw reads obtained from *C. tripartitus* using Illumina next-generation sequencer**

| Total number of raw reads |  |
| --- | --- |
| - Number of sequences | 51,207,282 |
| - Number of bases | 7,157,952,349 |
| Total read pairs processed | 25,603,641 |
| - Read 1 with adapter | 1,234,621 |
| - Read 2 with adapter | 1,108,154 |
| Pairs written (passing filters) | 25,603,641 |
| Total base pairs processed (bp) | 7,157,952,349 |
| - Read 1 (bp) | 3,580,581,011 |
| - Read 2 (bp) | 3,577,371,338 |
| Total written (filtered) (bp) | 7,146,338,412 |
| - Read 1 (bp) | 3,573,253,098 |
| - Read 2 (bp) | 3,573,085,314 |
| average length after trimming (bp) | 139.6 |
| percent of reads discarded (%) | 0.2% |

| Program | Cutadapt |
| --- | --- |
| Adapter 1 sequence | AGATCGGAAGAGCACACGTCTGAACTCCAGTCAC |
| Adapter 2 sequence | AGATCGGAAGAGCGTCGTGTAGGGAAAGAGTGTAGATCTCGGTGGTCGCCGTATCATT |

**Table S2: Classification of *C. tripartitus* Candidate genes to the innate immune signaling process**

| Candidate genes | Unigenes ID | | Length (bp) |
| --- | --- | --- | --- |
| PRR pathogen recognition receptor | | | |
| Immune signalling pathway | | | |
| Toll-like receptor 2 | Ct_Uni_02302, Ct_Uni_02303, Ct_Uni_10510, Ct_Uni_12097, Ct_Uni_16676, Ct_Uni_22346, Ct_Uni_24759 | | 1355, 2686, 2816, 545, 532, 1724, 2616 |
| PREDICTED: toll-like receptor Tollo | Ct_Uni_08072, Ct_Uni_13122 | | 2286, 1534 |
| PREDICTED: Toll-like receptor 2 | Ct_Uni_12547 | | 774 |
| PREDICTED: tolloid-like protein 1 | Ct_Uni_10258 | | 4119 |
| Neuropilin and tolloid-like protein 2 | Ct_Uni_22134 | | 648 |
| PREDICTED: neuropilin and tolloid-like protein 2 isoform X3 | Ct_Uni_12320 | | 1656 |
| Tollo | Ct_Uni_12765 | | 1643 |
| PREDICTED: toll-like receptor 6 | Ct_Uni_14535 | | 1554 |
| PREDICTED: toll-like receptor 7 | Ct_Uni_14678 | | 599 |
| PREDICTED: protein toll | Ct_Uni_07563 | | 3206 |
| Peptidoglycan recognition protein 3 short class | Ct_Uni_11324, Ct_Uni_14670, Ct_Uni_21140, Ct_Uni_21277 | | 695, 1690, 895, 755 |
| Peptidoglycan recognition protein-2 | Ct_Uni_11974 | | 926 |
| Peptidoglycan-recognition protein-SC2 | Ct_Uni_15467 | | 1094 |
| PREDICTED: peptidoglycan-recognition protein LC-like | Ct_Uni_07988, Ct_Uni_07989, Ct_Uni_18960, Ct_Uni_22619 | | 1565, 1130, 1584, 992 |
| PREDICTED: peptidoglycan-recognition protein LE isoform X1 | Ct_Uni_22028 | | 1279 |
| PREDICTED: peptidoglycan-recognition protein LE isoform X2 | Ct_Uni_08302 | | 1150 |
| PREDICTED: peptidoglycan-recognition protein SC2-like | Ct_Uni_12574 | | 789 |
| PREDICTED: peptidoglycan-recognition protein LA isoform X1 | Ct_Uni_22309 | | 1358 |
| PREDICTED: peptidoglycan-recognition protein LB isoform X1 | Ct_Uni_20876 | | 784 |
| C-type lectin | Ct_Uni_11004, Ct_Uni_11037 | | 1612, 1651 |
| C-type lectin, partial | Ct_Uni_12893, Ct_Uni_18991 | | 1001, 1629 |
| PREDICTED: CTL-like protein 2 isoform X1 | Ct_Uni_03781 | | 4669 |
| PREDICTED: CTL-like protein 2 isoform X3 | Ct_Uni_03782, Ct_Uni_03783, Ct_Uni_03784, Ct_Uni_03785 | | 4068, 4747, 4854, 4175 |
| PREDICTED: CTL-like protein 1 | Ct_Uni_07246 | | 2223 |
| Fibronectin domain-containing protein, partial | Ct_Uni_03656, Ct_Uni_14470, Ct_Uni_16514, Ct_Uni_16712, Ct_Uni_17587, Ct_Uni_18681, Ct_Uni_19991, Ct_Uni_21511, Ct_Uni_21705 | | 2043, 1873, 391, 230, 288, 3962, 3316, 2859, 460 |
| Fibronectin domain-containing protein | Ct_Uni_08873, Ct_Uni_08874, Ct_Uni_11741, Ct_Uni_13168, Ct_Uni_15159, Ct_Uni_15663, Ct_Uni_20141, Ct_Uni_22330 | | 5183, 5518, 3139, 1025, 1745, 9028, 2654, 4175 |
| Carbohydrate-binding protein | Ct_Uni_10417, Ct_Uni_12117, Ct_Uni_13020, Ct_Uni_14895, Ct_Uni_19826, Ct_Uni_20604, Ct_Uni_21730 | | 922, 2383, 1178, 2046, 2301, 2240, 802 |
| Carbohydrate-binding protein, partial | Ct_Uni_10619, Ct_Uni_11012, Ct_Uni_11193, Ct_Uni_13151 | | 4501, 1333, 1029, 1152 |
| PREDICTED: protein spaetzle-like | Ct_Uni_13893 | | 884 |
| PREDICTED: beta-1,3-glucan-binding protein 2 | Ct_Uni_11181 | | 992 |
| Galactoside-binding lectin | Ct_Uni_14508 | | 410 |
| PREDICTED: endoplasmic reticulum lectin 1 | Ct_Uni_19400 | | 1510 |
| PREDICTED: endoplasmic reticulum-Golgi intermediate compartment protein 3 isoform X1 | Ct_Uni_18649 | | 1517 |
| Chitinase | Ct_Uni_12074, Ct_Uni_18499, Ct_Uni_06865 | | 3132, 1566, 705 |
| PREDICTED: probable endochitinase | Ct_Uni_03016 | | 1666 |
| PREDICTED: endochitinase EP3-like | Ct_Uni_13398, Ct_Uni_20859, Ct_Uni_21134 | | 1162, 764, 847 |
| PREDICTED: probable chitinase 2 | Ct_Uni_15608, Ct_Uni_22778, Ct_Uni_02578, Ct_Uni_02579, Ct_Uni_02580, Ct_Uni_02581, Ct_Uni_02583, Ct_Uni_02584, Ct_Uni_02585, Ct_Uni_02586, Ct_Uni_02587, Ct_Uni_02588, Ct_Uni_02589, Ct_Uni_02590, Ct_Uni_06864, Ct_Uni_09230, Ct_Uni_09231 | | 1351, 1229, 3017, 3100, 2749, 2924, 2964, 2675, 2500, 3000, 2825, 3139, 2956, 2827, 1189, 2578, 2898 |
| PREDICTED: probable chitinase 3 | Ct_Uni_20152 | | 3359 |
| PREDICTED: probable chitinase 3, partial | Ct_Uni_03830, Ct_Uni_03832 | | 824, 1214 |
| PREDICTED: acidic mammalian chitinase-like | Ct_Uni_22327, Ct_Uni_06480 | | 1251, 1194 |
| PREDICTED: chitinase-like protein Idgf4 isoform X2 | Ct_Uni_04958, Ct_Uni_04959 | | 1648, 456 |
| PREDICTED: chitinase domain-containing protein 1 | Ct_Uni_05763 | | 1453 |
| Plectin, partial | Ct_Uni_10559, Ct_Uni_13336 | | 1021, 733 |
| PREDICTED: galectin-8-like | Ct_Uni_15340, Ct_Uni_08041, Ct_Uni_08042 | | 1563, 1658, 1437 |
| Galectin-4 | Ct_Uni_18478 | | 582 |
| Galectin-8 | Ct_Uni_21578 | | 558 |
| PREDICTED: galectin-4-like isoform X1 | Ct_Uni_23065 | | 1139 |
| PREDICTED: galectin-6-like isoform X2 | Ct_Uni_24577 | | 730 |
| PREDICTED: scavenger receptor class B member 1 | Ct_Uni_23140 | | 850 |
| PREDICTED: scavenger receptor class B member 1-like | Ct_Uni_15715, Ct_Uni_15773, Ct_Uni_16239, Ct_Uni_17583 | | 542, 342, 361, 267 |
| Scavenger receptor protein | Ct_Uni_07950 | | 1913 |
| PREDICTED: scavenger receptor class B, member 1-like isoform X1 | Ct_Uni_10413, Ct_Uni_12747 | | 1680, 2308 |
| Scavenger receptor class B, member 1-like | Ct_Uni_10796 | | 1732 |
| PREDICTED: Down syndrome cell adhesion molecule-like protein Dscam2 isoform X4 | Ct_Uni_08313 | | 1726 |
| PREDICTED: mucin-5AC | Ct_Uni_15660, Ct_Uni_17549, Ct_Uni_17839 | | 457, 263, 614 |
| PREDICTED: mucin-5AC isoform X7 | Ct_Uni_20105 | | 2456 |
| PREDICTED: mucin-5AC isoform X4 | Ct_Uni_03502, Ct_Uni_24109 | | 2561, 2635 |
| PREDICTED: mucin-5AC isoform X3 | Ct_Uni_04284 | | 4257 |
| PREDICTED: mucin-5AC-like, partial | Ct_Uni_13327 | | 484 |
| PREDICTED: mucin-22 isoform X1 | Ct_Uni_15576 | | 3881 |
| Insect intestinal mucin 3 | Ct_Uni_04171, Ct_Uni_04172, Ct_Uni_04173, Ct_Uni_04174 | | 4715, 2490, 1284, 1150 |
| Insect intestinal mucin 2 | Ct_Uni_08503 | | 333 |
| Intestinal mucin, partial | Ct_Uni_17505 | | 453 |
| PREDICTED: integumentary mucin C.1, partial | Ct_Uni_12616 | | 1298 |
| PREDICTED: mucin-2-like | Ct_Uni_01801, Ct_Uni_05649, Ct_Uni_07946, Ct_Uni_16815, Ct_Uni_24287 | | 767, 661, 1782, 489, 1507 |
| PREDICTED: mucin-2-like, partial | Ct_Uni_02840, Ct_Uni_14099 | | 3040, 1191 |
| PREDICTED: mucin-2-like isoform X2 | Ct_Uni_02841, Ct_Uni_02843, Ct_Uni_05650 | | 891, 1960, 942 |
| PREDICTED: mucin-2 | Ct_Uni_03083, Ct_Uni_17780 | | 838, 458 |
| Mucin-like protein, partial | Ct_Uni_02960 | | 577 |
| PREDICTED: mucin-17-like | Ct_Uni_17019 | | 373 |
| PREDICTED: mucin-19-like | Ct_Uni_02457 | | 671 |
| PREDICTED: mucin-12-like | Ct_Uni_16952 | | 497 |
| PREDICTED: mucin-12-like, partial | Ct_Uni_21779 | | 819 |
| PREDICTED: LOW QUALITY PROTEIN: mucin-17 | Ct_Uni_14135 | | 753 |
| PREDICTED: mucin-4 | Ct_Uni_16375 | | 426 |
| GNBP1 | Ct_Uni_18742 | | 1560 |
| PREDICTED: alpha-2-macroglobulin receptor-associated protein | Ct_Uni_12310 | | 1269 |
| TLR Signaling pathway | | | |
| Adaptor proteins | | | |
| PREDICTED: tumor necrosis factor alpha-induced protein 8-like protein isoform X1 | Ct_Uni_08641 | | 1796 |
| PREDICTED: tumor necrosis factor alpha-induced protein 8-like protein isoform X2 | Ct_Uni_08642 | | 2033 |
| PREDICTED: lipopolysaccharide-induced tumor necrosis factor-alpha factor-like | Ct_Uni_20862 | | 783 |
| PREDICTED: lipopolysaccharide-induced tumor necrosis factor-alpha factor homolog | Ct_Uni_21308 | | 798 |
| Sterile alpha and TIR motif-containing protein 1-like Protein | Ct_Uni_14294 | | 5475 |
| PREDICTED: sterile alpha and TIR motif-containing protein 1 isoform X3 | Ct_Uni_02916 | | 4923 |
| PREDICTED: sterile alpha and TIR motif-containing protein 1 isoform X2 | Ct_Uni_02917, Ct_Uni_02918 | | 4705, 8899 |
| MYD88 dependent pathway | | | |
| PREDICTED: pro-interleukin-16 | | Ct_Uni_07943 | 2286 |
| PREDICTED: interleukin enhancer-binding factor 2 homolog | | Ct_Uni_20120 | 1570 |
| PREDICTED: TGF-beta-activated kinase 1 and MAP3K7-binding protein 1-like | | Ct_Uni_19923, Ct_Uni_19972, Ct_Uni_20420 | 2802, 2751, 2010 |
| PREDICTED: TGF-beta receptor type-1 isoform X2 | | Ct_Uni_05727 | 7991 |
| PREDICTED: nuclear factor NF-kappa-B p110 subunit isoform X1 | | Ct_Uni_07644, Ct_Uni_07645 | 2905, 3020 |
| Inhibitor of nuclear factor kappa-B kinase subunit beta-like Protein | | Ct_Uni_12115 | 2414 |
| PREDICTED: transcription factor Adf-1-like | | Ct_Uni_10763 | 1237 |
| PREDICTED: transcription factor AP-4 | | Ct_Uni_12356 | 4555 |
| Interferon regulatory factor 2-binding protein 2-B | | Ct_Uni_19852, Ct_Uni_14866 | 3276, 2488 |
| PREDICTED: signal transducer and activator of transcription 5B isoform X1 | | Ct_Uni_24723 | 4214 |
| Mitogen-activated protein kinase kinase kinase 12 | | Ct_Uni_05402, Ct_Uni_05403 | 4388, 4066 |
| Mitogen-activated protein kinase ERK-A | | Ct_Uni_14771 | 2636 |
| Dual specificity mitogen-activated protein kinase kinase 3 | | Ct_Uni_03579, Ct_Uni_03580, Ct_Uni_03581, Ct_Uni_03582, Ct_Uni_03583 | 1702, 3087, 3182, 2982, 3097 |
| PREDICTED: dual specificity mitogen-activated protein kinase kinase 4 isoform X2 | | Ct_Uni_20345, Ct_Uni_20677 | 1952, 1985 |
| PREDICTED: mitogen-activated protein kinase kinase kinase kinase 5 isoform X7 | | Ct_Uni_05019 | 3384 |
| PREDICTED: mitogen-activated protein kinase kinase kinase kinase 5 isoform X2 | | Ct_Uni_05017, Ct_Uni_05018 | 1458, 3462 |
| PREDICTED: mitogen-activated protein kinase kinase kinase kinase 5 isoform X5 | | Ct_Uni_05016 | 3405 |
| PREDICTED: mitogen-activated protein kinase kinase kinase kinase 5 isoform X4 | | Ct_Uni_24278 | 1408 |
| PREDICTED: mitogen-activated protein kinase kinase kinase 4 isoform X1 | | Ct_Uni_03543 | 4467 |
| PREDICTED: mitogen-activated protein kinase kinase kinase 4 isoform X2 | | Ct_Uni_03540 | 4313 |
| PREDICTED: mitogen-activated protein kinase kinase kinase 4 | | Ct_Uni_03541 | 1205 |
| PREDICTED: mitogen-activated protein kinase kinase kinase 15 isoform X1 | | Ct_Uni_06588, Ct_Uni_06590 | 4371, 4437 |
| Mitogen-activated protein kinase ERK-A | | Ct_Uni_14771 | 2636 |
| Dual specificity mitogen-activated protein kinase kinase hemipterous-like Protein | | Ct_Uni_20213 | 1919 |
| PREDICTED: MAP kinase-activated protein kinase 2 | | Ct_Uni_01955, Ct_Uni_01957 | 1783, 553 |
| Cactin-like Protein | | Ct_Uni_08616 | 2333 |
| PREDICTED: Protein pellino | | Ct_Uni_20293 | 2030 |
| Suppressor of cytokine signaling 6 | | Ct_Uni_22172 | 1061 |
| PREDICTED: suppressor of cytokine signaling 2-like | | Ct_Uni_08650, Ct_Uni_08651, Ct_Uni_08652 | 3998, 4015, 3908 |
| Suppressor of cytokine signaling 5-like protein | | Ct_Uni_20028 | 2934 |
| PREDICTED: NF-kappa-B inhibitor cactus | | Ct_Uni_20553 | 2145 |
| PREDICTED: inhibitor of nuclear factor kappa-B kinase subunit epsilon | | Ct_Uni_20098 | 2540 |
| PREDICTED: serpin B12 | | Ct_Uni_20570 | 2121 |
| Serpin 15, partial | | Ct_Uni_23902, Ct_Uni_02179 | 590, 515 |
| PREDICTED: serpin B6-like isoform X11 | | Ct_Uni_02161 | 718 |
| Serpin 7, partial | | Ct_Uni_02162 | 299 |
| Serpin-2 | | Ct_Uni_14157, Ct_Uni_15890 | 608, 357 |
| PREDICTED: serpin B4-like isoform X2 | | Ct_Uni_15747 | 1578 |
| Serpin peptidase inhibitor 6 | | Ct_Uni_17658 | 352 |
| ENDOGENOUS LIGANDS | | | |
| Heat shock protein 23, partial | | Ct_Uni_21031 | 725 |
| heat shock protein 67B2 | | Ct_Uni_22925 | 661 |
| Heat shock protein 70, hsp70A2, partial | | Ct_Uni_21674 | 552 |
| Heat shock protein 67B2 | | Ct_Uni_22925 | 661 |
| PREDICTED: heat shock protein 68-like | | Ct_Uni_24443, Ct_Uni_24444 | 1659, 1603 |
| Heat shock protein 70 | | Ct_Uni_01867, Ct_Uni_01868, Ct_Uni_02459, Ct_Uni_06596 | 3685, 2486, 1243, 1606 |
| Heat shock protein 68a | | Ct_Uni_02458, Ct_Uni_02461, Ct_Uni_10963 | 4515, 2162, 681 |
| Hsp70, partial | | Ct_Uni_02460 | 635 |
| Heat shock protein cognate 4, partial | | Ct_Uni_07477, Ct_Uni_15443, Ct_Uni_18169 | 498, 727, 265 |
| Heat shock protein 90 | | Ct_Uni_07584, Ct_Uni_11776, Ct_Uni_16586 | 912, 2631, 345 |
| Heat shock protein 70 kDa, partial | | Ct_Uni_08407 | 387 |
| Heat shock cognate 71 kDa protein, putative | | Ct_Uni_06595 | 1901 |
| Heat shock protein 68-like Protein | | Ct_Uni_12890 | 2719 |
| Heat shock protein 90, partial | | Ct_Uni_14766, Ct_Uni_16156 | 549, 396 |
| Hsp90 co-chaperone | | Ct_Uni_10634 | 2340 |
| Heat shock protein Hsp-12.2 | | Ct_Uni_15995 | 532 |
| Heat shock cognate 70 | | Ct_Uni_01549, Ct_Uni_05799, Ct_Uni_07478 | 925, 487, 2547 |
| Heat shock cognate 70-like protein, partial | | Ct_Uni_17225 | 259 |
| Heat shock 70 kDa protein cognate | | Ct_Uni_05798 | 616 |
| PREDICTED: heat shock 70 kDa protein cognate 2-like | | Ct_Uni_19815 | 2191 |
| PREDICTED: heat shock 70 kDa protein 4 | | Ct_Uni_20002 | 3155 |
| PREDICTED: heat shock 70 kDa protein 4 isoform X1 | | Ct_Uni_19953 | 3194 |
| Heat shock protein 70 cognate, partial | | Ct_Uni_16217 | 281 |
| Heat shock protein cognate 4, partial | | Ct_Uni_18169 | 265 |
| Heat shock protein 60 | | Ct_Uni_17256 | 563 |
| Heat shock protein 70A, partial | | Ct_Uni_18410 | 392 |
| Small heat shock protein | | Ct_Uni_11770 | 751 |
| PREDICTED: heat shock protein 83-like isoform 1 | | Ct_Uni_07585 | 1611 |
| PREDICTED: heat shock 70 kDa protein cognate 5 | | Ct_Uni_09158 | 4354 |
| PREDICTED: HSPB1-associated protein 1 | | Ct_Uni_22032 | 1248 |
| Hsp90 co-chaperone | | Ct_Uni_10634 | 2340 |
| PREDICTED: HSPB1-associated protein 1 isoform X2 | | Ct_Uni_11503 | 1331 |
| Small heat shock protein | | Ct_Uni_18449 | 306 |
| PREDICTED: cold shock domain-containing protein 3-like | | Ct_Uni_18255 | 245 |
| PREDICTED: cold shock domain-containing protein E1 | | Ct_Uni_21784 | 4741 |
| PREDICTED: cold shock domain-containing protein E1-like | | Ct_Uni_18467, Ct_Uni_22724 | 478, 836 |
| Chaperone | | Ct_Uni_06318, Ct_Uni_06319, Ct_Uni_06320, Ct_Uni_11870 | 1916, 1992, 1515, 2568 |
| FK506-binding like-protein | | Ct_Uni_24570 | 1288 |
| PREDICTED: FK506-binding protein 59 | | Ct_Uni_18602, Ct_Uni_18920 | 1609, 1755 |
| PREDICTED: FK506-binding protein 5-like | | Ct_Uni_10601 | 3010 |
| PREDICTED: 12 kDa FK506-binding protein-like | | Ct_Uni_15402 | 312 |
| PREDICTED: stress-induced-phosphoprotein 1-like | | Ct_Uni_15123 | 401 |
| Stress-activated protein kinase JNK | | Ct_Uni_20378 | 3715 |
| Hypoxia-inducible factor 1-alpha, partial | | Ct_Uni_12191, Ct_Uni_12907 | 2374, 2863 |
| PREDICTED: hypoxia-inducible factor 1-alpha | | Ct_Uni_12192, Ct_Uni_12908 | 3086, 3575 |
| PREDICTED: prolyl 3-hydroxylase 1-like | | Ct_Uni_12479 | 1269 |
| PREDICTED: prolyl 3-hydroxylase sudestada1 | | Ct_Uni_18846 | 1782 |
| PREDICTED: CREB-binding protein isoform X3 | | Ct_Uni_18824 | 3721 |
| PREDICTED: CREB-binding protein isoform X6 | | Ct_Uni_18838 | 3691 |
| PREDICTED: TAF5-like RNA polymerase II p300/CBP-associated factor-associated factor 65 kDa subunit 5L | | Ct_Uni_23064, Ct_Uni_12486 | 963, 1864 |
| IMMUNE EFFECTORS | | | |
| PREDICTED: superoxide dismutase [Cu-Zn]-like | | Ct_Uni_13423 | 637 |
| Superoxide dismutase (Cu-Zn), partial | | Ct_Uni_20827 | 716 |
| Superoxide dismutase 2 | | Ct_Uni_22779 | 928 |
| PREDICTED: extracellular superoxide dismutase [Cu-Zn]-like | | Ct_Uni_11224 | 517 |
| PREDICTED: copper chaperone for superoxide dismutase | | Ct_Uni_21215 | 906 |
| PREDICTED: catalase-like | | Ct_Uni_24538, Ct_Uni_07635, Ct_Uni_09755, Ct_Uni_17733 | 264, 1617, 626, 613 |
| PREDICTED: catalase | | Ct_Uni_20740 | 1789 |
| PREDICTED: glutathione peroxidase 1-like | | Ct_Uni_13675, Ct_Uni_21350 | 869, 861 |
| PREDICTED: nitric oxide synthase-interacting protein homolog | | Ct_Uni_13932, Ct_Uni_13995 | 1045, 1048 |
| Sestrin | | Ct_Uni_20225 | 3552 |
| PREDICTED: thioredoxin domain-containing protein 9 | | Ct_Uni_08858 | 1602 |
| Thioredoxin domain-containing protein 5 | | Ct_Uni_09065, Ct_Uni_09066 | 1493, 1424 |
| Thioredoxin | | Ct_Uni_10346, Ct_Uni_10971, Ct_Uni_12671, Ct_Uni_14712, Ct_Uni_19222, Ct_Uni_19233, Ct_Uni_19893, Ct_Uni_03100, Ct_Uni_07024, Ct_Uni_07025 | 2210, 1032, 1168, 1629, 4154, 4219, 4013, 1095, 361, 1001 |
| Thioredoxin, partial | | Ct_Uni_18611, Ct_Uni_19266, Ct_Uni_19979, Ct_Uni_22399 | 1593, 1230, 799, 1175 |
| PREDICTED: thioredoxin domain-containing protein 15 | | Ct_Uni_22981 | 989 |
| Thioredoxin domain-containing protein 17-like Protein | | Ct_Uni_24201, Ct_Uni_24202, Ct_Uni_04193, Ct_Uni_04194 | 1656, 1708, 1521, 1412 |
| PREDICTED: thioredoxin reductase 1, mitochondrial isoform X4 | | Ct_Uni_04401, Ct_Uni_04402, Ct_Uni_04403, Ct_Uni_04404 | 4210, 4965, 4965, 3918 |
| PREDICTED: thioredoxin domain-containing protein 11 isoform X2 | | Ct_Uni_09584 | 3201 |
| PREDICTED: thioredoxin-like protein 4A | | Ct_Uni_21647 | 564 |
| PREDICTED: thioredoxin-like protein 1 | | Ct_Uni_22427 | 1157 |
| PREDICTED: peroxiredoxin 1-like | | Ct_Uni_08886 | 827 |
| PREDICTED: peroxiredoxin-6-like | | Ct_Uni_16081, Ct_Uni_22708 | 295, 894 |
| PREDICTED: peroxiredoxin-6 | | Ct_Uni_22193 | 1150 |
| PREDICTED: peroxiredoxin-1 | | Ct_Uni_22692, Ct_Uni_23084 | 970, 992 |
| PREDICTED: glutaredoxin-related protein 5, mitochondrial | | Ct_Uni_11361 | 701 |
| PREDICTED: glutaredoxin domain-containing cysteine-rich protein CG12206-like | | Ct_Uni_14268 | 2185 |
| Glutathione S-transferase, partial | | Ct_Uni_14686, Ct_Uni_21138, Ct_Uni_08962 | 393, 912, 1640 |
| Glutathione S-transferases-2 | | Ct_Uni_15359, Ct_Uni_16296 | 541, 296 |
| PREDICTED: glutathione S-transferase 1, isoform C-like | | Ct_Uni_15615, Ct_Uni_21274 | 669, 802 |
| Glutathione S-transferase | | Ct_Uni_16340, Ct_Uni_16431, Ct_Uni_20162, Ct_Uni_20791, Ct_Uni_20924, Ct_Uni_21300, Ct_Uni_21315, Ct_Uni_22153, Ct_Uni_22584, Ct_Uni_09115, Ct_Uni_10357, Ct_Uni_10358, Ct_Uni_12489, Ct_Uni_12887, Ct_Uni_13447, Ct_Uni_13762, Ct_Uni_14100 | 374, 668, 2659, 727, 836, 892, 828, 665, 889, 1090, 1225, 1193, 839, 1867, 891, 960, 967 |
| Glutathione S-transferases-1 | | Ct_Uni_17306, Ct_Uni_17903 | 600, 346 |
| PREDICTED: glutathione S-transferase omega-1-like | | Ct_Uni_17409 | 251 |
| PREDICTED: glutathione S-transferase | | Ct_Uni_18491, Ct_Uni_21144, Ct_Uni_03285, Ct_Uni_03286, Ct_Uni_07227, Ct_Uni_07228 | 1530, 875, 800, 1013, 560, 443 |
| PREDICTED: glutathione S-transferase 1-like | | Ct_Uni_18643, Ct_Uni_21019, Ct_Uni_21174, Ct_Uni_10138 | 1480, 809, 1012, 1586 |
| Glutathione S-transferase sigma | | Ct_Uni_21169, Ct_Uni_24505, Ct_Uni_07219, Ct_Uni_13444, Ct_Uni_13902 | 995, 382, 768, 798, 820 |
| Glutathione S-transferase epsilon | | Ct_Uni_21237, Ct_Uni_00466 | 809, 947 |
| PREDICTED: glutathione peroxidase 1-like | | Ct_Uni_21350, Ct_Uni_13675 | 861, 869 |
| PREDICTED: glutathione S-transferase theta-1 | | Ct_Uni_23036, Ct_Uni_13486 | 1022, 922 |
| PREDICTED: glutathione synthetase-like isoform X1 | | Ct_Uni_10200 | 1903 |
| PREDICTED: glutathione synthetase-like isoform X2 | | Ct_Uni_10201 | 1864 |
| Glutathione S-transferase C-terminal domain-containing protein | | Ct_Uni_12137 | 2478 |
| putative glutathione s-transferase, partial | | Ct_Uni_13795 | 831 |
| PREDICTED: glutathione S-transferase Mu 1-like | | Ct_Uni_13436 | 636 |
| PREDICTED: evolutionarily conserved signaling intermediate in Toll pathway, mitochondrial | | Ct_Uni_19365, Ct_Uni_19709 | 1531, 2426 |
| PREDICTED: cathepsin O-like | | Ct_Uni_20381, Ct_Uni_22478 | 1767, 1335 |
| Cathepsin L | | Ct_Uni_21716, Ct_Uni_24637, Ct_Uni_11303, Ct_Uni_17718 | 520, 296, 1277,440 |
| PREDICTED: cathepsin L1-like | | Ct_Uni_22033, Ct_Uni_12749, Ct_Uni_15994, Ct_Uni_17862 | 1216, 960, 467, 248 |
| PREDICTED: cathepsin L-like | | Ct_Uni_23918, Ct_Uni_24658, Ct_Uni_02319, Ct_Uni_02321, Ct_Uni_02322, Ct_Uni_02325, Ct_Uni_02326, Ct_Uni_02327, Ct_Uni_02328, Ct_Uni_09040, Ct_Uni_09837, Ct_Uni_12994, Ct_Uni_13302, Ct_Uni_16271, Ct_Uni_16593, Ct_Uni_17142, Ct_Uni_17212, Ct_Uni_17274, Ct_Uni_17506, Ct_Uni_18288 | 896, 244, 1088, 1066, 1087, 789, 1017, 1043, 931, 984, 646, 1074, 423, 330, 510, 343, 286, 330, 600, 643 |
| Cathepsin L, partial | | Ct_Uni_02320 | 1026 |
| Cathepsin L3 | | Ct_Uni_02323 | 241 |
| PREDICTED: cathepsin J-like | | Ct_Uni_05446, Ct_Uni_07202, Ct_Uni_07203,  Ct_Uni_09353, Ct_Uni_13828, Ct_Uni_18036, Ct_Uni_18315 | 1040, 560, 930, 1030, 1018, 564, 352 |
| PREDICTED: cathepsin L | | Ct_Uni_08623, Ct_Uni_11271, Ct_Uni_14296 | 1342, 550, 1370 |
| PREDICTED: cathepsin S-like | | Ct_Uni_09795, Ct_Uni_10893, Ct_Uni_16035 | 1031, 703, 485 |
| PREDICTED: cathepsin L1 | | Ct_Uni_10243 | 1241 |
| PREDICTED: cathepsin B | | Ct_Uni_10610 | 1397 |
| Cathepsin L-like protein | | Ct_Uni_14414 | 1078 |
| Cathepsin o | | Ct_Uni_16857 | 295 |
| C1 family cathepsin L11 | | Ct_Uni_20251 | 2016 |
| Putative cathepsin l | | Ct_Uni_16216 | 475 |
| PREDICTED: cathepsin L-like isoform 1 | | Ct_Uni_16672 | 743 |
| PREDICTED: cathepsin L-like isoform 4 | | Ct_Uni_17057 | 453 |
| Prophenoloxidase-II | | Ct_Uni_19809, Ct_Uni_19867 | 2231, 2312 |
| Prophenoloxidase-I | | Ct_Uni_19848 | 2292 |
| Prophenoloxidase activating factor | | Ct_Uni_20291 | 2014 |
| Pro-phenoloxidase activating enzyme-I precursor | | Ct_Uni_12965 | 1467 |
| Prophenoloxidase activating factor-III | | Ct_Uni_12966 | 2159 |
| ANTIMICROBIAL PEPTIDES | | | |
| PREDICTED: lysozyme 2-like | | Ct_Uni_12283 | 628 |
| AF425264_1 lysozyme precursor | | Ct_Uni_18464 | 337 |
| Lysozyme P-like Protein | | Ct_Uni_21508, Ct_Uni_21612 | 556, 568 |
| Antibacterial peptide Cp1 | | Ct_Uni_01652, Ct_Uni_01655, Ct_Uni_01656, Ct_Uni_08827, Ct_Uni_25093, Ct_Uni_25094 | 653, 284, 294, 269, 957, 943 |
| Antibacterial peptide Cp6 | | Ct_Uni_06088, Ct_Uni_06089 | 683, 476 |
| Coleoptericin | | Ct_Uni_04862, Ct_Uni_04864 | 948, 557 |
| Coprisin | | Ct_Uni_11389, Ct_Uni_16231, Ct_Uni_16627, Ct_Uni_17655, Ct_Uni_21565 | 329, 457, 506, 397, 943 |
| Holotricin 2 precursor | | Ct_Uni_13007, Ct_Uni_16195, Ct_Uni_21418, Ct_Uni_04920, Ct_Uni_04921, Ct_Uni_04922 | 535, 459, 604, 500, 318, 882 |
| Defensin 1, partial | | Ct_Uni_16067 | 400 |
| Thaumatin-like protein | | Ct_Uni_21289 | 793 |
| CYTOKINES AND CYTOKINE RECEPTORS | | | |
| PREDICTED: macrophage migration inhibitory factor homolog | | Ct_Uni_13736 | 499 |
| PREDICTED: lymphokine-activated killer T-cell-originated protein kinase | | Ct_Uni_10980 | 1270 |
| PREDICTED: pro-interleukin-16 | | Ct_Uni_07943 | 2286 |
| PREDICTED: T-cell immunomodulatory protein | | Ct_Uni_19753 | 2105 |
| OTHERS | | | |
| PREDICTED: septin-7 | | Ct_Uni_14998 | 2782 |
| PREDICTED: septin-2 | | Ct_Uni_18490 | 1630 |
| PREDICTED: apolipophorins | | Ct_Uni_11002 | 10200 |
| Apolipoprotein D | | Ct_Uni_11161, Ct_Uni_22526, Ct_Uni_05220 | 658, 902, 846 |
| Apolipoprotein D, partial | | Ct_Uni_12995, Ct_Uni_08489 | 464, 871 |
| PREDICTED: apolipoprotein D-like | | Ct_Uni_21450, Ct_Uni_22604, Ct_Uni_23002, Ct_Uni_05223 | 587, 1017, 1275, 280 |
| PREDICTED: apolipoprotein O-like | | Ct_Uni_22571 | 933 |
| PREDICTED: apolipoprotein D | | Ct_Uni_22659, Ct_Uni_03095 | 924, 674 |
| Apolipoprotein D-like precursor | | Ct_Uni_22915 | 666 |
| Putative apolipoprotein d/lipocalin, partial | | Ct_Uni_14141 | 628 |
| PREDICTED: 14-3-3 protein zeta isoform X2 | | Ct_Uni_08529 | 1630 |
| PREDICTED: 14-3-3 protein zeta-like | | Ct_Uni_08382, Ct_Uni_15942 | 885, 466 |
| QM protein | | Ct_Uni_21673 | 375 |
| Lipoprotein receptor | | Ct_Uni_02237, Ct_Uni_02247, Ct_Uni_08672, Ct_Uni_08673, Ct_Uni_10349, Ct_Uni_19554, Ct_Uni_22177 | 1493, 1687, 1070, 997, 1396, 1413, 1123 |
| Lipoprotein receptor, partial | | Ct_Uni_12538, Ct_Uni_15077 | 5672, 378 |
| Low-density lipoprotein receptor-related protein 2 | | Ct_Uni_18934 | 1699 |
| PREDICTED: low-density lipoprotein receptor-related protein 2-like | | Ct_Uni_16245, Ct_Uni_18121 | 226, 394 |
| PREDICTED: low-density lipoprotein receptor-related protein 6 | | Ct_Uni_09980 | 5052 |
| PREDICTED: low-density lipoprotein receptor-related protein 2 isoform X1 | | Ct_Uni_03946, Ct_Uni_17703 | 11784, 404 |
| PREDICTED: low-density lipoprotein receptor-related protein 4 isoform X2 | | Ct_Uni_04925, Ct_Uni_04926 | 1932, 7353 |
| PREDICTED: very low-density lipoprotein receptor isoform X3 | | Ct_Uni_19877 | 3276 |
| PREDICTED: very low-density lipoprotein receptor isoform X2 | | Ct_Uni_20591, Ct_Uni_20603 | 3360, 3384 |
| PREDICTED: beclin-1-like protein A | | Ct_Uni_03977, Ct_Uni_08240, Ct_Uni_08241, Ct_Uni_21527 | 839, 1098, 1095, 738 |
| PREDICTED: target of rapamycin | | Ct_Uni_06400 | 7427 |
| CD63 antigen | | Ct_Uni_19535, Ct_Uni_19626 | 1354, 1677 |
| PREDICTED: CD63 antigen | | Ct_Uni_10270 | 770 |
| Chitinase | | Ct_Uni_06865, Ct_Uni_12074, Ct_Uni_18499 | 705, 3132, 1566 |
| PREDICTED: probable chitinase 2 | | Ct_Uni_02578, Ct_Uni_02579, Ct_Uni_02580, Ct_Uni_02581, Ct_Uni_02583, Ct_Uni_02584, Ct_Uni_02585, Ct_Uni_02586, Ct_Uni_02587, Ct_Uni_02588, Ct_Uni_02589, Ct_Uni_02590, Ct_Uni_06864, Ct_Uni_09230, Ct_Uni_09231, Ct_Uni_15608, Ct_Uni_22778 | 3017, 3100, 2749, 2924, 2964, 2675, 2500, 3000, 2825, 3139, 2956, 2827, 1189, 2578, 2898, 1351, 1229 |
| PREDICTED: probable endochitinase | | Ct_Uni_03016 | 1666 |
| PREDICTED: probable chitinase 3, partial | | Ct_Uni_03830, Ct_Uni_03832 | 824, 1214 |
| PREDICTED: chitinase-like protein Idgf4 isoform X2 | | Ct_Uni_04958, Ct_Uni_04959 | 1648, 456 |
| PREDICTED: chitinase domain-containing protein 1 | | Ct_Uni_05763 | 1453 |
| PREDICTED: endochitinase EP3-like | | Ct_Uni_13398, Ct_Uni_20859, Ct_Uni_21134 | 1162, 764, 847 |
| PREDICTED: probable chitinase 3 | | Ct_Uni_20152 | 3359 |
| PREDICTED: calreticulin-like | | Ct_Uni_14315, Ct_Uni_16212 | 493, 537 |
| PREDICTED: tetraspanin-5 isoform X2 | | Ct_Uni_06906, Ct_Uni_06907 | 1747, 1652 |
| PREDICTED: tetraspanin-5 isoform X1 | | Ct_Uni_06908, Ct_Uni_24477, Ct_Uni_25053, Ct_Uni_25054 | 2958, 1688, 1581, 1486 |
| PREDICTED: tetraspanin-3-like isoform X2 | | Ct_Uni_17795 | 374 |
| Tetraspannin | | Ct_Uni_11373, Ct_Uni_18909, Ct_Uni_19033, Ct_Uni_19624, Ct_Uni_20540 | 1128, 1614, 1632, 1080, 1734 |
| Tetraspannin, partial | | Ct_Uni_14059, Ct_Uni_22535 | 1114, 974 |
| PREDICTED: tetraspanin-7-like | | Ct_Uni_18810 | 1663 |
| PREDICTED: tetraspanin-9 | | Ct_Uni_21968 | 1151 |
| PREDICTED: tetraspanin-9-like | | Ct_Uni_22242 | 1773 |
| APOPTOSIS | | | |
| PREDICTED: TNF receptor-associated factor 6 | | Ct_Uni_11397 | 1541 |
| PREDICTED: TNF receptor-associated factor 4 isoform X1 | | Ct_Uni_14618 | 1781 |
| PREDICTED: lipopolysaccharide-induced tumor necrosis factor-alpha factor-like | | Ct_Uni_20862 | 783 |
| PREDICTED: lipopolysaccharide-induced tumor necrosis factor-alpha factor homolog | | Ct_Uni_21308 | 798 |
| Apoptosis regulator BAX | | Ct_Uni_22185 | 1157 |
| PREDICTED: apoptosis inhibitor 5 | | Ct_Uni_10624 | 2566 |
| PREDICTED: apoptosis-inducing factor 3 | | Ct_Uni_14335 | 1798 |
| PREDICTED: caspase-7-like | | Ct_Uni_01908, Ct_Uni_01909, Ct_Uni_11710, Ct_Uni_15332 | 1297, 1075, 1863, 1756 |
| Caspase | | Ct_Uni_06329 | 1349 |
| PREDICTED: caspase-1-like | | Ct_Uni_13609 | 947 |
| Caspase Nc-like | | Ct_Uni_15036 | 2698 |
| Caspase-3 | | Ct_Uni_21976 | 1116 |
| PREDICTED: baculoviral IAP repeat-containing protein 6 isoform X2 | | Ct_Uni_04870 | 11747 |
| PREDICTED: baculoviral IAP repeat-containing protein 2-like | | Ct_Uni_14585 | 904 |
| PREDICTED: baculoviral IAP repeat-containing protein 5 | | Ct_Uni_16109 | 545 |
| PREDICTED: esterase B1-like | | Ct_Uni_07646, Ct_Uni_07647, Ct_Uni_13667 | 3954, 2484, 891 |
| PREDICTED: esterase E4-like | | Ct_Uni_20373, Ct_Uni_20535 | 1772, 1719 |
| Esterase FE4 | | Ct_Uni_18046, Ct_Uni_18053 | 439, 439 |
| PREDICTED: esterase FE4 | | Ct_Uni_00236, Ct_Uni_00585, Ct_Uni_00586, Ct_Uni_00587, Ct_Uni_00750, Ct_Uni_00751, Ct_Uni_07599, Ct_Uni_23324, Ct_Uni_23325, Ct_Uni_23373, Ct_Uni_23374 | 2400, 2164, 1959, 1959, 2035, 1804, 1450, 1507, 1859, 1504, 1127 |
| Esterase | | Ct_Uni_00419, Ct_Uni_09308, Ct_Uni_12255, Ct_Uni_12827, Ct_Uni_20232 | 802, 2456, 549, 1938, 2030 |
| Esterase, partial | | Ct_Uni_15767 | 339 |
| Esterase 6 precursor | | Ct_Uni_20252 | 1994 |
| Putative esterase | | Ct_Uni_00600, Ct_Uni_00601, Ct_Uni_04424, Ct_Uni_04425, Ct_Uni_04426, Ct_Uni_07018, Ct_Uni_07019, Ct_Uni_07088, Ct_Uni_07615, Ct_Uni_08074, Ct_Uni_10606, Ct_Uni_11905, Ct_Uni_14675, Ct_Uni_24648, Ct_Uni_24649 | 2269, 2218, 3719, 3667, 3725, 1613, 1677, 1726, 968, 1653, 2082, 2077, 1683, 1574, 1495 |
| putative esterase, partial | | Ct_Uni_10895 | 1294 |
| PREDICTED: venom carboxylesterase-6-like | | Ct_Uni_01883, Ct_Uni_01884, Ct_Uni_01887, Ct_Uni_05766, Ct_Uni_05767, Ct_Uni_18382 | 509, 694, 2104, 1768, 1262, 448 |
| PREDICTED: venom carboxylesterase-6 | | Ct_Uni_01885, Ct_Uni_04886, Ct_Uni_04887, Ct_Uni_04888, Ct_Uni_23007 | 511, 3668, 3736, 1875, 1191 |
| PREDICTED: venom carboxylesterase-6-like isoform X2 | | Ct_Uni_17375 | 498 |
| Carboxylesterase | | Ct_Uni_01889, Ct_Uni_01890, Ct_Uni_07087, Ct_Uni_07622, Ct_Uni_10884, Ct_Uni_09751 | 722, 783, 1795, 1420, 555, 1853 |
| Rel A | | Ct_Uni_02833, Ct_Uni_02834, Ct_Uni_02835, Ct_Uni_04991, Ct_Uni_09763, Ct_Uni_23993, Ct_Uni_23994, Ct_Uni_24704 | 2597, 1898, 2514, 776, 650, 1224, 804, 396 |
| PREDICTED: apoptotic protease-activating factor 1 isoform X1 | | Ct_Uni_07842 | 4253 |
| PREDICTED: apoptosis regulatory protein Siva-like | | Ct_Uni_11890 | 540 |
| PREDICTED: inhibitor of apoptosis 2 isoform X1 | | Ct_Uni_14341 | 1736 |
| Programmed cell death protein 2-like | | Ct_Uni_14552 | 1565 |
| PREDICTED: programmed cell death protein 4-like | | Ct_Uni_15814, Ct_Uni_16588 | 292, 359 |
| PREDICTED: p53 and DNA damage-regulated protein 1 | | Ct_Uni_16747 | 594 |
| PREDICTED: programmed cell death protein 2 | | Ct_Uni_19058 | 1424 |
| PREDICTED: programmed cell death 6-interacting protein | | Ct_Uni_20038 | 2876 |
| TP53-regulated inhibitor of apoptosis 1-like Protein | | Ct_Uni_21212, Ct_Uni_22799 | 854, 792 |
| PREDICTED: programmed cell death protein 10 | | Ct_Uni_22176 | 1356 |
| AUTOPHAGY | | | |
| PREDICTED: autophagy-related protein 16-1 isoform X2 | | Ct_Uni_06536 | 1603 |
| PREDICTED: autophagy-related protein 2 homolog B isoform X1 | | Ct_Uni_24596 | 5264 |
| PREDICTED: autophagy-related protein 2 homolog A isoform X3 | | Ct_Uni_08245, Ct_Uni_08246 | 6485, 6420 |
| PREDICTED: autophagy-related protein 13 homolog isoform X1 | | Ct_Uni_09362 | 2598 |
| PREDICTED: beclin 1-associated autophagy-related key regulator isoform X2 | | Ct_Uni_14563 | 1555 |
| Autophagy-related protein 3 | | Ct_Uni_19543 | 1369 |
| Autophagy-related protein 10 | | Ct_Uni_15683 | 310 |
| Autophagy-related protein 13 | | Ct_Uni_21389 | 671 |
| DNA damage-regulated autophagy modulator protein 2, partial | | Ct_Uni_17770 | 251 |
| PREDICTED: autophagy-related protein 101 | | Ct_Uni_24755 | 2823 |
| PREDICTED: ubiquitin-like protein ATG12 | | Ct_Uni_22862 | 1571 |

**Table S3: Genes of interest related to growth in the dung beetle, *C. tripartitus***

| Candidate genes | Unigenes ID | Length (bp) |
| --- | --- | --- |
| Somatotrophic axis | | |
| PREDICTED: insulin receptor-like isoform X3 | Ct_Uni_05876 | 3197 |
| PREDICTED: insulin-like peptide receptor | Ct_Uni_06640, Ct_Uni_24449, Ct_Uni_24451 | 663, 360, 497 |
| PREDICTED: insulin gene enhancer protein ISL-1 isoform X3 | Ct_Uni_06881 | 2113 |
| Insulin receptor, partial | Ct_Uni_07735 | 5447 |
| PREDICTED: insulin-like growth factor 2 mRNA-binding protein 1 isoform X5 | Ct_Uni_07977 | 6278 |
| Insulin-like growth factor 2 mRNA-binding protein 1 | Ct_Uni_07978 | 6360 |
| PREDICTED: insulin receptor substrate 1 isoform X10 | Ct_Uni_08891 | 2858 |
| PREDICTED: insulin-like growth factor-binding protein complex acid labile subunit | Ct_Uni_08924, Ct_Uni_19611 | 393, 1064 |
| PREDICTED: insulin-like growth factor-binding protein complex acid labile subunit isoform X10 | Ct_Uni_10206 | 950 |
| PREDICTED: insulin-degrading enzyme | Ct_Uni_10360 | 6834 |
| Epidermal growth factor receptor substrate 15-like 1 | Ct_Uni_20083 | 4046 |
| PREDICTED: 5-hydroxytryptamine receptor isoform X1 | Ct_Uni_17768 | 295 |
| PREDICTED: 5-hydroxytryptamine receptor 1D-like, partial | Ct_Uni_18041 | 292 |
| PREDICTED: 5-hydroxytryptamine receptor 1A-like | Ct_Uni_20558 | 2088 |
| PREDICTED: 5-hydroxytryptamine receptor 1 isoform X1 | Ct_Uni_20980 | 706 |
| PREDICTED: cysteine-rich protein 1-like | Ct_Uni_21383 | 576 |
| PREDICTED: adenosine deaminase CECR1-A-like | Ct_Uni_14604 | 1522 |
| PREDICTED: adenosine deaminase CECR1 | Ct_Uni_18857 | 1790 |
| PREDICTED: POU domain, class 6, transcription factor 2-like, partial | Ct_Uni_06275, Ct_Uni_06276 | 3379, 3876 |
| PREDICTED: POU domain, class 6, transcription factor 1 isoform X3 | Ct_Uni_06277 | 2638 |
| PREDICTED: POU domain protein CF1A | Ct_Uni_08678, Ct_Uni_08679 | 2062, 2244 |
| PREDICTED: POU domain, class 6, transcription factor 2 | Ct_Uni_12967 | 1928 |
| PREDICTED: PAX-interacting protein 1-like isoform X2 | Ct_Uni_21689 | 374 |
| PREDICTED: PAX-interacting protein 1 | Ct_Uni_04785, Ct_Uni_04786 | 4310, 2298 |
| Muscle growth | | |
| Actin binding protein, partial | Ct_Uni_22453, Ct_Uni_24064, Ct_Uni_24065, Ct_Uni_24066, Ct_Uni_24067, Ct_Uni_24068, Ct_Uni_24069, Ct_Uni_03326, Ct_Uni_03327, Ct_Uni_03328, Ct_Uni_03329, Ct_Uni_03330, Ct_Uni_03331, Ct_Uni_03332, Ct_Uni_03333, Ct_Uni_10094, Ct_Uni_15596, Ct_Uni_16762, Ct_Uni_16951, Ct_Uni_17330, Ct_Uni_17392, Ct_Uni_21356, Ct_Uni_21543 | 7775, 3525, 3654, 3753, 6295, 6549, 4045, 3750, 6166, 3879, 6205, 5893, 6335, 6449, 6426, 12436, 769, 7634, 7520, 715, 502, 771, 320 |
| Actin-5C, partial | Ct_Uni_23952, Ct_Uni_02539, Ct_Uni_16782 | 245, 799, 396 |
| Actin 5C/42A, partial | Ct_Uni_23953 | 405 |
| Actin binding protein | Ct_Uni_24190, Ct_Uni_02638, Ct_Uni_02639, Ct_Uni_02640, Ct_Uni_02641, Ct_Uni_02642, Ct_Uni_04120, Ct_Uni_04122, Ct_Uni_09368,  Ct_Uni_21192 | 302, 4685, 5005, 2152, 5715, 5668, 5079, 12590, 755, 951 |
| PREDICTED: actin-binding LIM protein 1 isoform X5 | Ct_Uni_25059, Ct_Uni_25060, Ct_Uni_25065, Ct_Uni_25066 | 3836, 3425, 3905, 3494 |
| Actin, partial | Ct_Uni_02538, Ct_Uni_06485, Ct_Uni_18450 | 713, 569, 287 |
| Actin-binding LIM protein 1 | Ct_Uni_11376, Ct_Uni_11377, Ct_Uni_14702 | 3647, 4053, 3983 |
| PREDICTED: actin-binding protein IPP | Ct_Uni_12099 | 4770 |
| PREDICTED: actin-binding LIM protein 1 isoform X7 | Ct_Uni_12832, Ct_Uni_12833 | 3864, 3453 |
| PREDICTED: actin-related protein 8 | Ct_Uni_14468 | 1934 |
| PREDICTED: actin-binding protein anillin-like | Ct_Uni_14803, Ct_Uni_20546 | 3149, 1756 |
| PREDICTED: actin-binding LIM protein 3 isoform X4 | Ct_Uni_14974 | 2854 |
| PREDICTED: actin-related protein 5 | Ct_Uni_15176 | 2006 |
| PREDICTED: actin-related protein 2/3 complex subunit 5 | Ct_Uni_15623 | 1431 |
| Actin-1, partial | Ct_Uni_15735, Ct_Uni_16784 | 301, 835 |
| PREDICTED: actin-like protein 6B | Ct_Uni_19023 | 1679 |
| PREDICTED: tropomyosin-1, isoforms 33/34 isoform X3 | Ct_Uni_02795 | 1137 |
| PREDICTED: tropomyosin-1, isoforms 33/34-like isoform X1 | Ct_Uni_05814 | 494 |
| Tropomyosin | Ct_Uni_11543, Ct_Uni_13526, Ct_Uni_17065 | 971, 752, 280 |
| Tropomyosin 1, partial | Ct_Uni_16325 | 629 |
| PREDICTED: tropomyosin-1, isoforms 9A/A/B isoform X27 | Ct_Uni_16619 | 591 |
| Tropomyosin-1, isoforms 9A/A/B | Ct_Uni_17459 | 477 |
| PREDICTED: tropomyosin-1 | Ct_Uni_19117 | 1380 |
| Tropomyosin 1 | Ct_Uni_19322 | 1273 |
| Miscellaneous | | |
| Chitinase | Ct_Uni_12074, Ct_Uni_18499, Ct_Uni_06865 | 3132, 1566, 705 |
| PREDICTED: endochitinase EP3-like | Ct_Uni_13398, Ct_Uni_20859, Ct_Uni_21134 | 1162, 764, 847 |
| PREDICTED: probable chitinase 2 | Ct_Uni_15608, Ct_Uni_22778, Ct_Uni_02578, Ct_Uni_02579, Ct_Uni_02580, Ct_Uni_02581, Ct_Uni_02583, Ct_Uni_02584, Ct_Uni_02585, Ct_Uni_02586, Ct_Uni_02587, Ct_Uni_02588, Ct_Uni_02589, Ct_Uni_02590, Ct_Uni_06864, Ct_Uni_09230, Ct_Uni_09231 | 1351, 1229, 3017, 3100, 2749, 2924, 2964, 2675, 2500, 3000, 2825, 3139, 2956, 2827, 1189, 2578, 2898 |
| PREDICTED: probable chitinase 3 | Ct_Uni_20152 | 3359 |
| PREDICTED: probable chitinase 3, partial | Ct_Uni_03830, Ct_Uni_03832 | 824, 1214 |
| PREDICTED: probable endochitinase | Ct_Uni_03016 | 1666 |
| PREDICTED: chitinase-like protein Idgf4 isoform X2 | Ct_Uni_04958, Ct_Uni_04959 | 1648, 456 |
| PREDICTED: chitinase domain-containing protein 1 | Ct_Uni_05763 | 1453 |
| collagen, partial | Ct_Uni_13885, Ct_Uni_15968, Ct_Uni_16519 | 5689, 5740, 367 |
| PREDICTED: collagen alpha-2(IV) chain isoform X2 | Ct_Uni_14884 | 990 |
| PREDICTED: collagen alpha-1(XII) chain-like isoform X2 | Ct_Uni_15802 | 437 |
| Collagen alpha-5(VI) chain/sw-like protein, partial | Ct_Uni_17260 | 398 |
| PREDICTED: collagen alpha-1(IX) chain-like isoform X8 | Ct_Uni_18379 | 469 |
| PREDICTED: collagen type IV alpha-3-binding protein isoform X1 | Ct_Uni_19756 | 2145 |
| PREDICTED: collagen alpha-1(IX) chain-like | Ct_Uni_02729 | 445 |
| PREDICTED: collagenase-like | Ct_Uni_04837 | 873 |
| Collagen alpha-2(IV) chain | Ct_Uni_06750 | 637 |
| PREDICTED: collagen alpha-1(XVIII) chain isoform X2 | Ct_Uni_08337 | 1902 |
| PREDICTED: soma ferritin-like | Ct_Uni_17828 | 649 |
| Ferritin | Ct_Uni_22005 | 1082 |
| PREDICTED: apolipophorins | Ct_Uni_11002 | 10200 |
| PREDICTED: DNA topoisomerase 2 isoform X1 | Ct_Uni_12681 | 4964 |
| PREDICTED: DNA topoisomerase 2-binding protein 1-B isoform X1 | Ct_Uni_13916 | 648 |
| PREDICTED: DNA topoisomerase 3-beta-1 | Ct_Uni_19921 | 3152 |
| PREDICTED: DNA topoisomerase 3-alpha | Ct_Uni_06209 | 3193 |
| PREDICTED: calcitonin gene-related peptide type 1 receptor-like | Ct_Uni_07940 | 1934 |

**Table-S4 Candidate Sex-Determination and Reproduction related genes from *C.* tripartitus unigenes**

| Candidate genes | Unigenes ID | Length (bp) |
| --- | --- | --- |
| PREDICTED: WD repeat and FYVE domain-containing protein 3 isoform X1 | Ct_Uni_03297, Ct_Uni_03298 | 7531, 10412 |
| PREDICTED: WD repeat-containing protein 24 | Ct_Uni_03669, Ct_Uni_03670, Ct_Uni_03671, Ct_Uni_03672 | 4402, 4447, 2896, 2851 |
| WD repeat, SAM and U-box domain-containing protein 1-like Protein | Ct_Uni_03891, Ct_Uni_03892, Ct_Uni_03893, Ct_Uni_03894, Ct_Uni_03895, Ct_Uni_03896 | 6581, 6650, 6454, 6389, 6532, 6328 |
| PREDICTED: WD repeat-containing protein 48 homolog isoform X1 | Ct_Uni_04968, Ct_Uni_04969, Ct_Uni_04970, Ct_Uni_04971 | 4270, 4210, 3207, 1312 |
| PREDICTED: glutamate-rich WD repeat-containing protein 1 | Ct_Uni_05195, Ct_Uni_05196, Ct_Uni_05197 | 1805, 2119, 2214 |
| PREDICTED: WD repeat-containing protein 47 isoform X3 | Ct_Uni_05241, Ct_Uni_05242, Ct_Uni_05243, Ct_Uni_05244 | 2542, 3582, 2398, 3958 |
| PREDICTED: WD repeat-containing protein 82 | Ct_Uni_05657 | 3125 |
| PREDICTED: WD repeat and HMG-box DNA-binding protein 1 isoform X1 | Ct_Uni_07209, Ct_Uni_07210 | 2957, 2812 |
| PREDICTED: putative transcription factor SOX-15 isoform X2 | Ct_Uni_09016 | 1752 |
| PREDICTED: transcription factor Sox-2 | Ct_Uni_09730, Ct_Uni_14612,  Ct_Uni_24924 | 2157,1914, 1524 |
| PREDICTED: transcription factor Sox-10 | Ct_Uni_11843 | 1668 |
| PREDICTED: transcription factor Sox-3 | Ct_Uni_20453 | 1871 |
| PREDICTED: transcription factor Sox-13 | Ct_Uni_08554 | 3397 |
| PREDICTED: transcription factor Sox-21-A | Ct_Uni_22308 | 1288 |
| PREDICTED: protein mab-21 | Ct_Uni_09667 | 1419 |
| PREDICTED: protein mab-21-like | Ct_Uni_16481, Ct_Uni_17197 | 692, 665 |
| PREDICTED: GATA zinc finger domain-containing protein 14-like isoform X4 | Ct_Uni_01762 | 2089 |
| PREDICTED: GATA zinc finger domain-containing protein 14-like isoform X5 | Ct_Uni_02995, Ct_Uni_16650 | 2261, 523 |
| PREDICTED: GATA zinc finger domain-containing protein 10-like | Ct_Uni_07033, Ct_Uni_07034 | 2847, 2931 |
| PREDICTED: transcription factor GATA-4 isoform X2 | Ct_Uni_09592, Ct_Uni_09593 | 2027, 2016 |
| PREDICTED: GATA zinc finger domain-containing protein 1 | Ct_Uni_14074 | 982 |
| PREDICTED: GATA-binding factor A isoform X1 | Ct_Uni_22140 | 646 |
| Sex-determining protein Fem-1 | Ct_Uni_01692 | 1238 |
| PREDICTED: sex determination protein fruitless isoform X4 | Ct_Uni_22278 | 1544 |
| PREDICTED: spermatogenesis-associated protein 13-like isoform X1 | Ct_Uni_03942, Ct_Uni_03944 | 1264, 3237 |
| PREDICTED: spermatogenesis-associated protein 13-like isoform X2 | Ct_Uni_08703 | 2238 |
| PREDICTED: spermatogenesis-associated protein 5 | Ct_Uni_09583 | 2436 |
| Spermatogenesis-associated protein 13-like protein | Ct_Uni_07355 | 3929 |
| PREDICTED: spermine oxidase-like | Ct_Uni_09996, Ct_Uni_14973, Ct_Uni_24730 | 2897, 2858, 2621 |
| PREDICTED: spermine synthase isoform X1 | Ct_Uni_19347 | 1421 |
| PREDICTED: spermine synthase isoform X2 | Ct_Uni_21995 | 1420 |
| PREDICTED: spermidine synthase | Ct_Uni_22592 | 949 |
| PREDICTED: round spermatid basic protein 1-like | Ct_Uni_23116 | 8486 |
| PREDICTED: testis-expressed sequence 2 protein isoform X1 | Ct_Uni_04392 | 1709 |
| PREDICTED: testis-expressed sequence 10 protein homolog | Ct_Uni_08704 | 2120 |
| Vitellogenin-1 | Ct_Uni_05212, Ct_Uni_05213, Ct_Uni_15232 | 5600, 4647, 400 |
| Vitellogenin-2 | Ct_Uni_05271, Ct_Uni_05272, Ct_Uni_05273, Ct_Uni_05274, Ct_Uni_24305, Ct_Uni_24306, Ct_Uni_24307 | 5595, 2234, 5571, 5591, 5604, 5578, 5594 |
| PREDICTED: vitellogenin-6-like | Ct_Uni_16443 | 430 |
| Vitellogenin receptor, partial | Ct_Uni_16973 | 578 |
| PREDICTED: vitellogenin-like | Ct_Uni_21190 | 5446 |
| Vitellogenin | Ct_Uni_07191, Ct_Uni_13240 | 1859, 359 |
| PREDICTED: putative vitellogenin receptor | Ct_Uni_05819, Ct_Uni_05820, Ct_Uni_11015, Ct_Uni_24364, Ct_Uni_24495 | 5738, 1390, 318, 2916, 740 |
| PREDICTED: sperm protamine P1-like | Ct_Uni_05214 | 890 |
| PREDICTED: sperm surface protein Sp17 | Ct_Uni_19314 | 1270 |
| Doublesex male-specific isoform | Ct_Uni_05482 | 1377 |
| Doublesex female-specific isoform type 2 | Ct_Uni_05483 | 2153 |
| Doublesex female-specific isoform type 1 | Ct_Uni_05484 | 2121 |
| PREDICTED: meiosis arrest female protein 1 isoform X3 | Ct_Uni_07685, Ct_Uni_07687 | 4124, 4017 |
| PREDICTED: meiosis arrest female protein 1 isoform X1 | Ct_Uni_07686 | 5041 |
| PREDICTED: follicle-stimulating hormone receptor-like isoform X1 | Ct_Uni_09602 | 2445 |
| PREDICTED: follicle-stimulating hormone receptor | Ct_Uni_17039 | 336 |
| PREDICTED: sex determination protein fruitless isoform X3 | Ct_Uni_11183 | 420 |
| PREDICTED: sex determination protein fruitless isoform X5 | Ct_Uni_11287 | 1431 |
| PREDICTED: motile sperm domain-containing protein 1-like | Ct_Uni_13611 | 913 |
| PREDICTED: motile sperm domain-containing protein 2 | Ct_Uni_20429 | 2439 |
| PREDICTED: dual specificity testis-specific protein kinase 2 | Ct_Uni_14919, Ct_Uni_14967, Ct_Uni_20077, Ct_Uni_24942, Ct_Uni_24954 | 3104, 3086, 3123, 3065, 3047 |
| Female-specific feminizer isoform f, partial | Ct_Uni_15225, Ct_Uni_20340, Ct_Uni_14324 | 2348,1902, 1700 |
| PREDICTED: spermatogenesis associated 6-like protein | Ct_Uni_15404 | 1448 |
| PREDICTED: gonadal protein gdl | Ct_Uni_17670 | 278 |
| PREDICTED: testis-specific serine/threonine-protein kinase 1-like | Ct_Uni_17940 | 717 |
| Transformer-2 sex-determining protein-like Protein | Ct_Uni_20113, Ct_Uni_24775 | 2460, 2166 |
